# Competing risks of major bleeding and thrombotic events with prasugrel-based dual antiplatelet therapy after stent implantation - An observational analysis from BASKET-PROVE II

**DOI:** 10.1371/journal.pone.0210821

**Published:** 2019-01-15

**Authors:** Raban V. Jeger, Matthias Pfisterer, Deborah R. Vogt, Søren Galatius, Ulrik Abildgaard, Christoph Naber, Hannes Alber, Franz Eberli, David J. Kurz, Giovanni Pedrazzini, André Vuilliomenet, Daniel Weilenmann, Hans Rickli, Kim Wadt Hansen, Peter Rickenbacher, David Conen, Christian Müller, Stefan Osswald, Nicole Gilgen, Christoph Kaiser

**Affiliations:** 1 Cardiology, University Hospital Basel, University Basel, Basel, Switzerland; 2 Cardiology, Gentofte University Hospital Copenhagen, Hellerup, Denmark; 3 Cardiology, Elisabeth-Krankenhaus, Essen, Germany; 4 Cardiology, University Hospital Innsbruck and Klinikum Klagenfurt am Wörthersee, Innsbruck and Klagenfurt am Wörthersee, Austria; 5 Cardiology, Triemli Hospital, Zürich, Switzerland; 6 Cardiology, Cardiocentro, Lugano, Switzerland; 7 Cardiology, State Hospital, Aarau, Switzerland; 8 Cardiology, State Hospital, St. Gallen, Switzerland; 9 Cardiology, State Hospital, Bruderholz, Switzerland; 10 Population Health Research Institute, McMaster University, Hamilton ON, Canada; Campus Bio-Medico University of Rome, ITALY

## Abstract

**Background:**

Dual antiplatelet therapy (DAPT) prevents thrombotic events after coronary stent implantation but may induce bleedings, specifically in elderly patients. However, a competitive risk analysis is lacking.

**Objectives:**

To assess the determinants of major bleeding and the balance between the competing risks of major bleeding and thrombotic events during prasugrel-based DAPT after stent implantation.

**Methods:**

Overall, 2,291 patients randomized to drug-eluting or bare metal stents and treated with prasugrel 10mg/day for 1 year were followed over 2 years for major bleeding (BARC 3/5) and thrombotic events (cardiac death, myocardial infarction, definitive/probable stent thrombosis). Prasugrel dose was reduced to 5mg in patients >75 years and/or <60kg. Predictors of major bleeding and competing risks of major bleeding and thrombotic events were assessed.

**Results:**

Two-year rates of major bleeding and thrombotic events were 2.9% and 9.0%, respectively. The only independent predictor of major bleeding was age (hazard ratio per year increase 1.05 [1.02,1.07], p<0.001). The relationship between major bleeding and age was non-linear, with lowest hazard ratios at 57 years and an exponential increase only above 65 years. In contrast, the relationship between thrombotic events and age was linear and continuously increasing with older age. While the competing risk of thrombotic events was higher than that of major bleeding in younger patients, the two risks were similar in older patients. After discontinuation of prasugrel, bleeding events leveled off in all patients, while thrombotic events continued to increase.

**Conclusions:**

In prasugrel-based DAPT, age is the strongest risk factor for major bleeding, increasing exponentially >65 years. In younger patients, thrombotic events represent a higher risk than bleeding, while thrombotic and bleeding risks were similar in older patients. Important clinical implications relate to prasugrel dose in the elderly, duration of DAPT and the competing risk balance necessitating individualized treatment decisions.

## Introduction

Dual antiplatelet therapy (DAPT) with aspirin and a platelet P_2_Y_12_ adenosine diphosphate receptor antagonist inhibits platelet aggregation and prevents recurrent ischemia and stent thrombosis. The combination of aspirin and clopidogrel has shown a clear benefit against aspirin alone regarding thrombotic events [1, 2] and entered guideline recommendations [3]. However, since up to one third of patients are low-responders or even resistant to clopidogrel [4], newer compounds such as prasugrel and ticagrelor have been developed [5, 6].

DAPT after percutaneous coronary intervention (PCI) entails an increased risk for major bleeding in both unselected patients [7] and patients with acute coronary syndromes (ACS) [8, 9]. Although prasugrel is superior to clopidogrel for ACS patients after PCI [10] based on the results of TRITON–TIMI 38 [6], it was associated with more bleedings, particularly in patients >75 years, and therefore, has not been recommended in this age group [11, 12]. However, a reduced dose of 5mg daily for patients <60kg was proposed based on ancillary studies of TRITON-TIMI 38 [13, 14] and entered label information of the European Medicines Agency [11]. Although actually not recommended for patients >75 years of age, prasugrel is used in this age group in many centers, mainly with the reduced-dose regimen of 5mg daily. The incidence of major bleeding in patients >75 years of age treated with low-dose prasugrel has been reported in a subgroup analysis of the TRILOGY-ACS study and was not different to clopidogrel [15]. However, the exact benefit-risk balance between the prevention of thrombotic events and the induction of major bleeding for prasugrel-based DAPT in different age groups is unknown.

In the multicenter BASKET-PROVE II trial, PCI patients were treated with bleeding-risk dose-adjusted prasugrel-based DAPT, irrespective of clinical indication and stent type [16]. Prasugrel was stopped after one year and patients followed for another 12 months. In this context, we performed a retrospective analysis of prospectively collected long-term findings to identify predictors of major bleeding and assess the competing risks of major bleeding and thrombotic events in the different age groups.

## Materials and methods

### Study design

**Fig 1:**
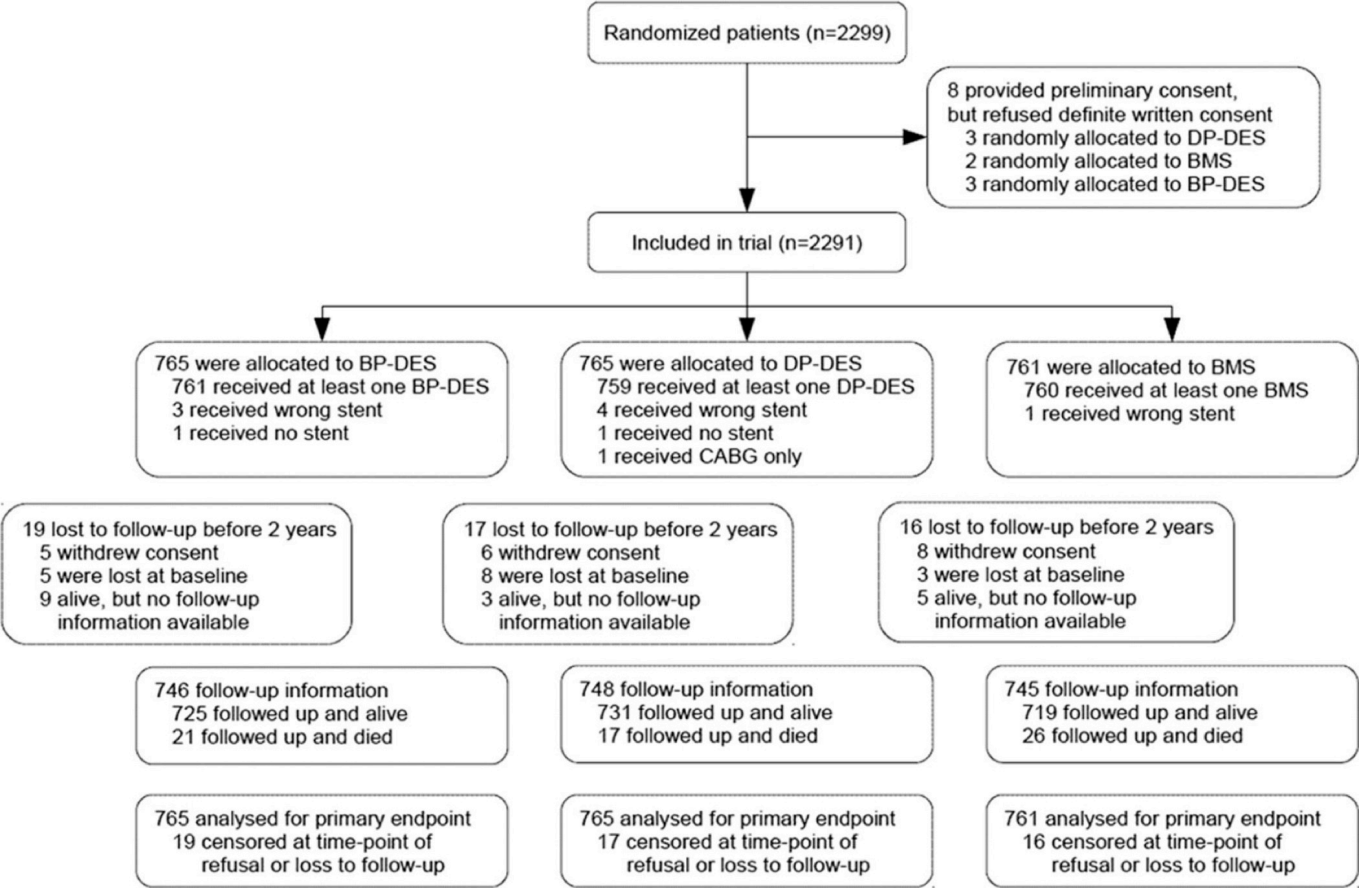
Patient flow chart. BMS indicates bare-metal stent; BP-DES, biodegradable-polymer drug-eluting stent; CABG, coronary artery bypass graft; and DP-DES: durable-polymer drug-eluting stent.

This is a retrospective, observational analysis of the randomized BASKET PROVE II trial that enrolled patients between April 2010 and May 2012. BASKET-PROVE II [16, 17] was a prospective, controlled, randomized multicenter trial that aimed to define the efficacy and safety of biodegradable-polymer biolimus A9-eluting stents (Nobori, Terumo, Somerset NJ) compared to durable-polymer everolimus-eluting stents (Xience Prime, Abbott Vascular, Abbott Park IL) and thin-strut silicone-carbide coated bare-metal stents (BMS; Prokinetik, Biotronik, Berlin, Germany) [17]. All patients received a prasugrel-based DAPT after stent implantation and were followed for two years. The patient flow chart is depicted in Fig 1.

Written informed consent was obtained from all patients. Elective patients were enrolled after giving written informed consent, while urgent or emergent patients first gave oral consent documented by a medical person not involved in the trial to avoid any time delay due to the enrolment procedure, and provided written informed consent after the intervention. The following Independent Ethics Committees were consulted: Ethikkommission beider Basel EKBB, Hebelstrasse 53, 4056 Basel, Switzerland; De Videnskabsetiske Komiteer I Region Hovedstaden, Kongens Vænge 2, 3400 Hillerød, Denmark; Ethikkommission der Medizinischen Universität Innsbruck, Innrain 43, 6020 Innsbruck, Austria; Ethikkommission der Ärztekammer Nordrhein, Tersteegenstrasse 9, 40474 Düsseldorf, Germany; Comitato etico cantonale, Via Orico 5, 6501 Bellinzona, Switzerland; Ethikkommission des Kantons St.Gallen, Kantonsspital/Flurhof 7, Rorschacherstrasse 95, 9007 St.Gallen, Switzerland; Kantonale Ethikkommission Zürich KEK (Abteilung 4), Sonneggstrasse 12, 8091 Zürich, Switzerland; Kantonale Ethikkommission Aargau, Bachstrasse 15, 5001 Aarau, Switzerland. The local ethics committees in each center approved the study protocol. The study was initially approved by the EKBB on November 20th, 2009. The first patient entered the study on April 1st, 2010, the last patient left the study on June 30th, 2014. The study complies with the Declaration of Helsinki.

The study was initially registered in the EUDRACT-registry on August 25th, 2009 before start of patient recruitment (EudraCT Number 2009-015616-18). In addition, the trial was registered in the clinicaltrials.gov registry before the study start. Unfortunately, it was registered twice under two different identifiers, i.e., under the project numbers NCT01097187 and NCT01166685 in November 2009. To avoid duplicity, the registration NCT01097187 (Record 2009-01516-18) was deleted and the registration NCT01166685 changed and released anew (July 2010).

### Patients

The study enrolled all-comer patients undergoing PCI with stents ≥3mm in diameter. Exclusion criteria included in-stent restenosis, stent thrombosis, bypass graft interventions, unprotected main stem lesions, cardiogenic shock, planned surgery within 12 months, oral anticoagulation, history of or active bleeding disorders, known hypersensitivity to antiplatelet medication, no follow-up possible, no compliance expected or any known cerebrovascular accident.

### Study procedures and treatment

PCI was performed according to standard techniques at the discretion of the interventional cardiologist in charge. If not already treated with the drugs, all patients received a loading dose of 250 to 500mg aspirin i.v. and 60mg prasugrel p.o., followed by a maintenance dose of 100mg aspirin and 10mg prasugrel p.o. daily over 12 months. Prasugrel was adjusted to the bleeding risk of patients, i.e., in patients >75 years of age and/or <60kg of body weight, prasugrel maintenance dose was reduced to 5mg p.o. [6]. Prasugrel was prescribed for 12 months in all ACS patients and/or patients receiving drug-eluting stents (DES) and for one month in stable coronary artery disease patients receiving BMS. To assure adherence to DAPT, patients were given the drug in monthly packages at regular intervals from the study centers. No further prasugrel was provided after 12 months. All patients were prescribed statin therapy. No routine angiographic follow-up was allowed unless clinically indicated.

### Endpoints and definitions

The primary endpoint for this analysis was time to major bleeding not related to coronary artery bypass graft surgery within 24±2 months, as defined according to the Bleeding Academic Research Consortium (BARC) [18] criteria 3 and 5. The main secondary endpoint was time to thrombotic events, i.e., cardiac death, non-fatal myocardial infarction, and probable/definite stent thrombosis, within 24±2 months. Cardiac death was defined as any death not clearly due to a non-cardiac cause. Myocardial infarction was defined as described previously [19] and stent thrombosis according to the Academic Research Consortium criteria [20].

### Statistical analyses

All analyses were performed according to the intention-to-treat principle. Baseline characteristics are reported as counts and percentages or means±standard deviation. Continuous variables were compared using Wilcoxon’s rank sum test, categorical variables using Fisher’s exact test. Time-to-event analyses were carried out, using the Kaplan-Meier estimator and Cox proportional hazards models, stratifying for center. The following variables were investigated as predictors for major bleeding: sex, age, weight, diabetes mellitus, renal disease, stent type, glycoprotein IIb/IIIa inhibitor use, prasugrel dose, and indication for PCI. In order to find the smallest combination of predictors for major bleeding, a backward model selection approach based on Akaike’s information criterion was applied. The selection process started with the maximal model including all of the above listed predictors plus the interaction term between age and prasugrel dose. No further interactions were considered. Only linear terms were explored. As first step, single predictors were removed step by step, until the Akaike’s information criterion did not decrease any further (automatic backwards selection). As second step, this model was further simplified by step-wise removal of the remaining predictors (manual backwards selection). Akaike’s information criterion was allowed to increase slightly, i.e. no more than 2 units for each removed predictor compared to the model with the minimal Akaike’s information criterion (Δ<2.0). No imputation of missing data was performed. For the model selection procedure only patients without any missing values in the predictor variables were used (n = 2232). The resulting final model was then refitted using all 2291 patients.

In order to explore the functional form of the relationship between major bleeding and age, several Cox models considering age as categorical or non-linear predictor were fit. Based on previous literature, age was initially categorized as <75 years and ≥75 years. Then, a non-linear relationship with age was explored. The observed proportions of events with 95% confidence intervals were plotted for different age groups (5-year classes, 10-year classes, and deciles) and explored visually. Finally, age was modeled more flexibly using natural cubic regression splines. The degrees of freedom, and thus the number and position of knots, were obtained by minimizing the Bayesian information criterion [21]. Pointwise nonparametric hazard ratios with 95% confidence intervals were estimated, and the smoothing hazard ratio curve with 95% confidence bands was shown for the observed age range.

For the competing risk analysis, thrombotic events were considered a competing risk to major bleeding, in the sense that the occurrence of a thrombotic event modifies the chances for the occurrence of major bleeding and vice versa. Therefore, we examined whether the effects of age differed between the two types of events or risks, i.e. were cause-specific. Cumulative incidence curves for each event type are shown overall and according to age dichotomized at 65 years. Estimates are based on the Aalen-Johansen estimator, an extension of the Kaplan-Meier estimator to the case of multiple, competing risks [22]. Patients who experienced the competing event, i.e. had a thrombotic event first, were censored at the time of occurrence of the competing event. Regression splines were fit as described before. Further, cause-specific hazards were compared between patients <65 and ≥65 years for each event and the relative hazards for the two competing events were compared within each age group by duplicating the data set (two rows per patient, one for each event type). The duplicated data set was then analyzed using a Cox proportional hazard model with event type and age group and their interaction as predictors. Model assumptions have been checked by inspection of the Schoenfeld residuals and deviance residuals.

All analyses were performed with the statistical software system R version 3.1.0.[23]. A p-value <0.05 indicated statistical significance.

## Results

Overall, 2291 patients were enrolled in BASKET PROVE II. The median age of all patients was 63.2 years (range 28.0 to 91.2 years), 77.7% were men, and 28.8% presented with ST-elevation myocardial infarction, 34.4% with Non-ST-elevation ACS and 36.9% with stable coronary disease [16]. Two-year rates of major bleeding and thrombotic events were 2.9% and 9.0%, respectively.

**Fig 2:**
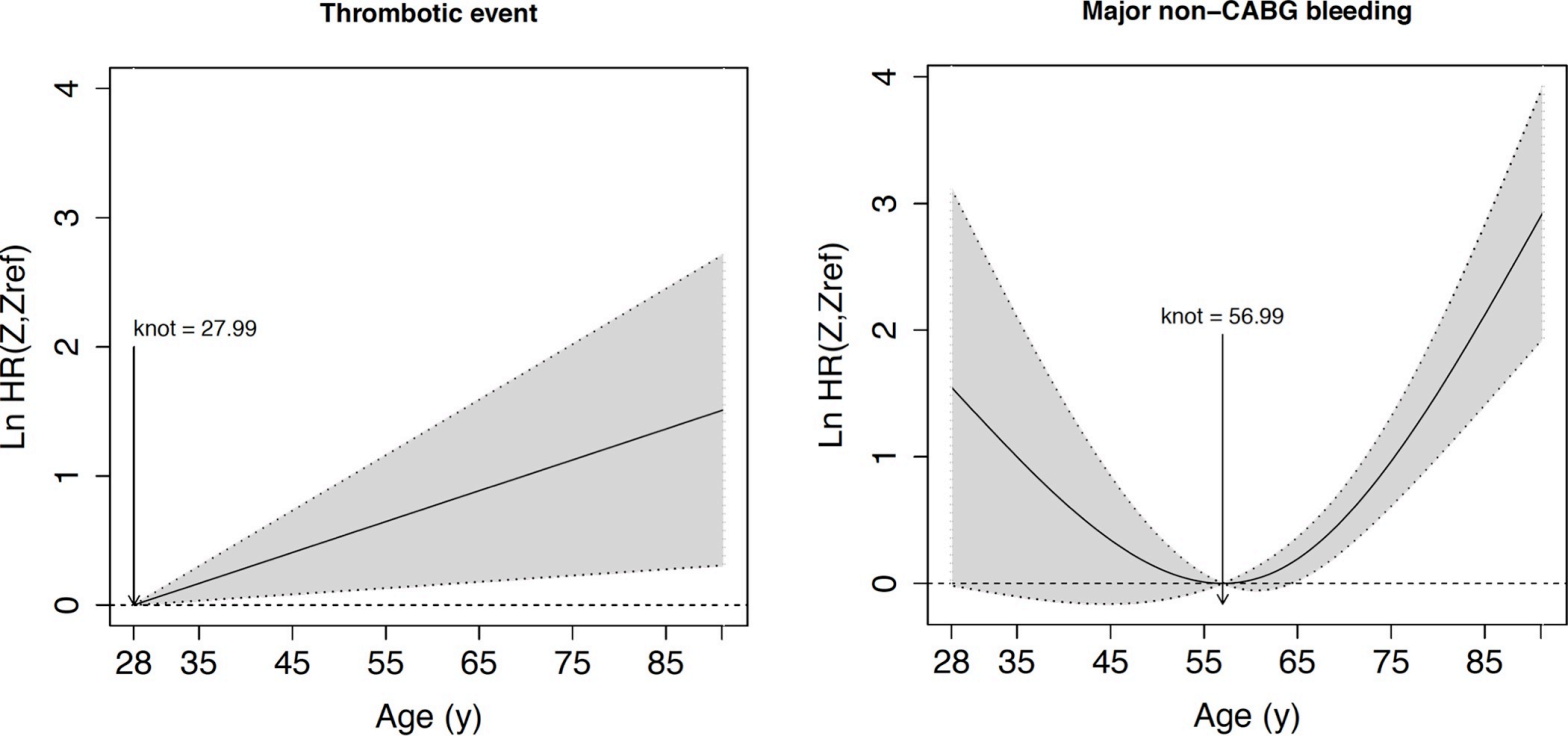
**Nonparametric estimates of the dependence of the case-specific hazard ratios for major non-CABG bleeding (Fig 2A) and thrombotic events (Fig 2B) on age.** Log hazard ratios with 95% confidence band are shown. The hazard ratios are relative to the age with the lowest hazard (major non-CABG bleeding: indicated knot = 57 years; thrombotic events: minimal age = 28 years). CABG, coronary artery bypass graft; LnHR, log hazard ratio; Z, specific value of age on x-axis; Zref, reference value of age with lowest hazard, i.e. indicated knot.

Age was the only independent predictor of major bleeding (hazard ratio 1.05 per year, 95% confidence interval 1.02, 1.07, p<0.001), while none of the other variables being assessed in the model reached statistical significance. The functional relationship of age with major bleeding showed a J-shaped curve with the lowest risk at the age of 57 years. Below 57 years, there was a somewhat higher risk for major bleeding, although the hazard ratio was not significantly increased (Fig 2A, Table 1). Above 57 years, there was a sharp and exponential increase in the risk of major bleeding that was statistically significant after the age of 65 years contrasting to the lack of a significant change in the risk below 57 years. The risk relation with age was different for thrombotic events with a steady increase with increasing age (Fig 2B, Table 1). Based on these findings patients were divided into younger and older than 65 years (Table 2). Baseline characteristics of these two groups showed that older patients were less frequently male and had a lower weight, suffered more often from diabetes mellitus and arterial hypertension but were less frequently smoking than younger patients <65 years. Older patients had undergone more prior revascularization procedures and had more known renal disease than younger patients. Older patients underwent PCI more frequently for stable angina and less frequently for ST-elevation MI than younger patients. Glycoprotein-IIb/IIIa-inhibitors were used less often in older than younger patients. The hazard ratio for major bleeding in patients older vs. younger than 65 years was 2.21 (95% confidence interval 1.33, 3.65, p<0.001).

**Table 1:** Baseline characteristics in patients <65 and ≥65 years of age.

	Patients <65 Years	Patients ≥65 Years	p
n	1286 (56.1)	1005 (43.9)	
**Demographics and medical history**			
Age (years)	55.0 (7.1)	73.1 (5.5)	<0.001
Sex (male)	1089 (84.7)	691 (68.8)	<0.001
Weight (kilogram)	85.0 (15.5)	79.5 (14.2)	<0.001
Diabetes mellitus	197 (15.3)	232 (23.1)	<0.001
- Insulin-dependent	50 (3.9)	60 (6.0)	0.027
Hypertension	766 (59.6)	757 (75.3)	<0.001
Hypercholesterolemia	812 (63.1)	637 (63.4)	0.940
Current smoking	653 (50.8)	165 (16.4)	<0.001
Renal disease	32 (2.5)	95 (9.5)	<0.001
Prior myocardial infarction	118 (9.2)	93 (9.3)	1.000
Prior PCI	154 (12.0)	153 (15.2)	0.028
Prior CABG	20 (1.6)	39 (3.9)	0.001
**Indication for PCI**			
Stable coronary artery disease	398 (30.9)	447 (44.5)	<0.001
Unstable angina pectoris and NSTEMI	450 (35.0)	337 (33.5)	0.493
STEMI	438 (34.1)	221 (22.0)	<0.001
**Coronary anatomy**			
Left main disease	6 (0.5)	7 (0.7)	0.655
Left anterior descending artery disease	799 (62.1)	650 (64.7)	0.226
Left circumflex disease	407 (31.6)	377 (37.5)	0.004
Right coronary artery disease	661 (51.4)	532 (52.9)	0.491
Multivessel disease	458 (35.6)	420 (41.8)	0.003
Bifurcation disease	67 (5.2)	54 (5.4)	0.940
Chronic total occlusion	56 (4.4)	30 (3.0)	0.109
**Percutaneous coronary intervention**			
Glycoprotein IIbIIIa inhibitor	204 (15.9)	80 (8.0)	<0.001
Stent per segment (n)	1.2 (0.5)	1.3 (0.6)	0.063
Stent per patient (n)	1.5 (0.8)	1.5 (0.8)	0.253
Stent length (mm)	25.9 (16.5)	26.2 (17.6)	0.677
Stent <3.0 mm	51 (4.0)	48 (4.8)	0.401
Max implantation pressure (atm)	15.0 (3.6)	14.5 (3.5)	<0.001
Staged procedure	80 (6.2)	67 (6.7)	0.733

PCI, percutaneous coronary intervention; CABG, coronary artery bypass graft; NSTEMI, non-ST-elevation myocardial infarction; STEMI, ST-elevation myocardial infarction. Baseline characteristics are reported as counts and percentages or means ± standard deviation. Continuous variables were compared using Wolcoxon’s rank sum test, categorical variables using Fisher’s exact test.

**Table 2:** Pointwise nonparametric estimation of cause-specific hazard ratios for major bleeding and thrombotic events as competing risks according to age.

Age	Major Bleeding			Thrombotic events		
	Hazard Ratio	95% Confidence Interval	Number of Events/ Patients	Hazard Ratio	95% Confindence Interval	Number of Events/ Patients
30	4.01	[0.96, 16.78]	0/2	1.05	[1.01, 1.09]	0/2
35	2.72	[0.9, 8.17]	2/9	1.19	[1.03, 1.35]	1/9
40	1.9	[0.86, 4.18]	0/31	1.34	[1.06, 1.68]	2/31
45	1.4	[0.85, 2.34]	1/84	1.51	[1.08, 2.08]	2/84
50	1.13	[0.87, 1.46]	4/183	1.7	[1.12, 2.59]	6/183
55	1.01	[0.95, 1.07]	4/265	1.92	[1.14, 3.19]	4/265
60	1.03	[0.95, 1.11]	5/336	2.16	[1.17, 3.97]	13/336
65	1.21	[1.02, 1.43]	7/379	2.44	[1.2, 4.9]	19/379
70	1.67	[1.31, 2.14]	8/351	2.72	[1.22, 6.11]	9/351
75	2.61	[1.84, 3.74]	8/304	3.06	[1.26, 7.54]	17/304
80	4.53	[2.72, 7.54]	15/220	3.46	[1.28, 9.3]	6/220
85	8.33	[4.1, 16.95]	8/105	3.9	[1.32, 11.59]	12/105
90	15.96	[6.23, 40.85]	2/22	4.39	[1.35, 14.3]	2/22

Hazard ratios are the ratios of the hazard for the respective age (e.g. 30 years) to the lowest hazards estimate (for major bleeding: 57 years; for thrombotic events: 28 years–c.f. Fig 2A and 2B). Number of Events/Patients give the number of observed first events and the number of patients in the respective age class (e.g. age 30 includes all patients < 30 years, age 35 includes all patients > = 30 years and < 35 years, age 90 includes all patients > 90 years). Please note that hazard ratios are point-wise model predictions, while number of events/patients summarize observations over age ranges.

**Fig 3:**
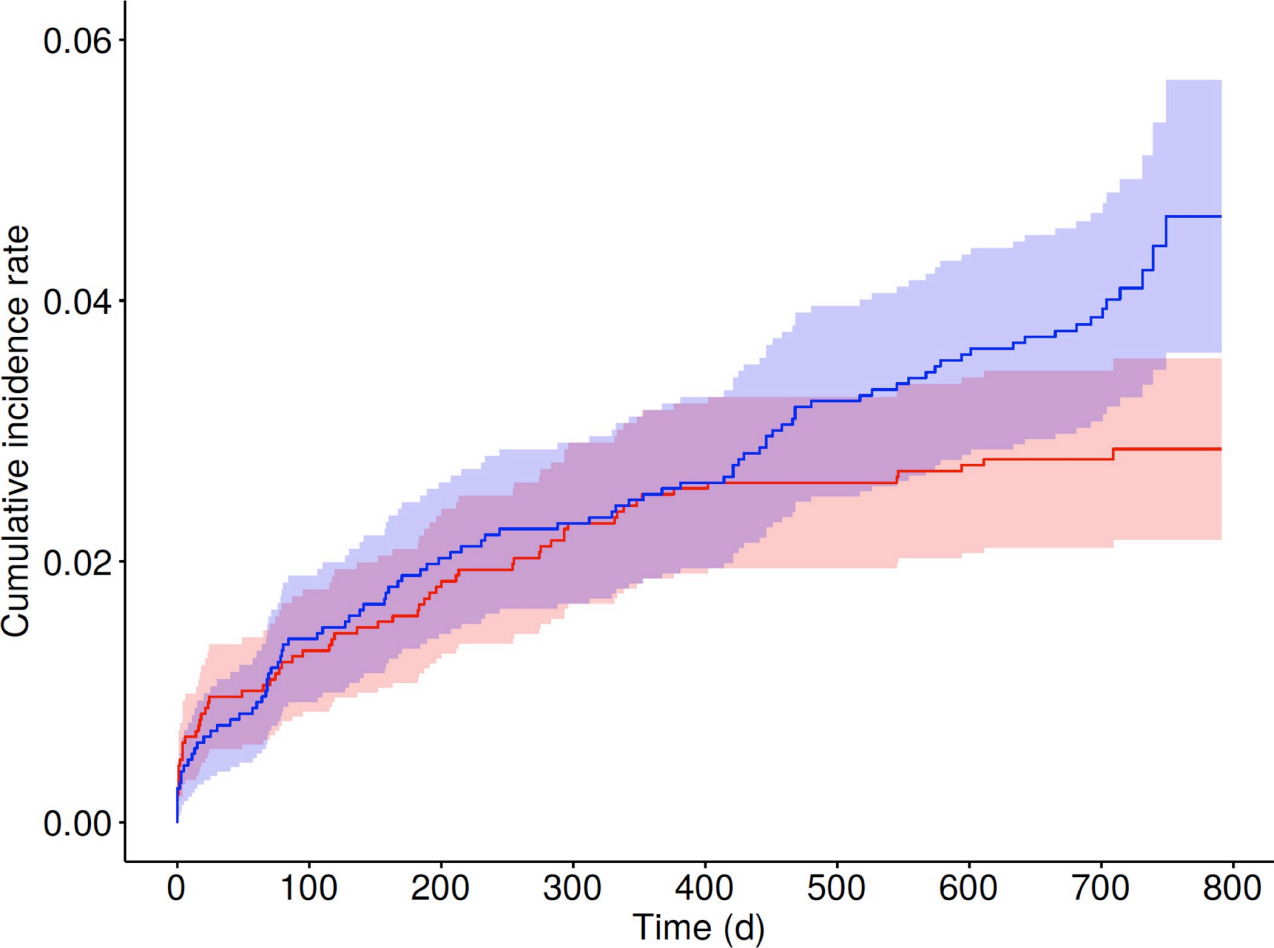
Cumulative incidence curves with 95% confidence bands for the competing events of non-CABG major bleeding (red) and thrombotic event (blue) in all 2,291 patients. CABG, coronary artery bypass graft.

**Fig 4:**
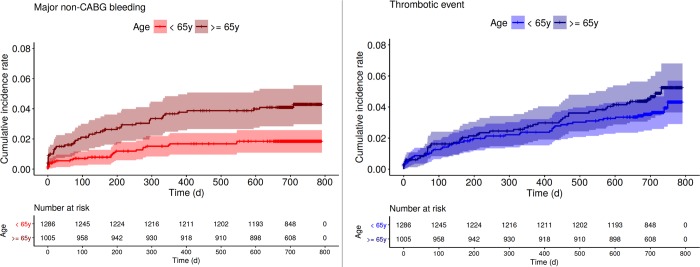
**Cumulative incidence curves with 95% confidence bands for the competing events of non-CABG major bleeding (Fig 4A) and thrombotic event (Fig 4B) according to age dichotomized at 65 years.** Note the significant differences in major bleedings indicated by the not overlapping confidence bands, but the non-significant differences in thrombotic events. CABG, coronary artery bypass graft.

Cumulative incidence curves for major bleeding and thrombotic events are shown in Fig 3. The rates of major bleeding increased during the initial year after stent implantation, i.e. during treatment with prasugrel and acetylsalicylic acid, but leveled off afterwards, i.e. after discontinuation of prasugrel, while rates of thrombotic events increased continuously until the end of the follow-up. A detailed analysis of these curves relative to the two age groups shows a persistently higher hazard for major bleeding events in patients ≥65 vs. <65 years of age during the entire follow-up (hazard ratio 2.34, 95% confidence interval 1.40, 3.90, p = 0.001, Fig 4A). In contrast, the hazard for thrombotic events between the two age groups were similar (hazard ratio 1.28, 95% confidence interval 0.85, 1.93, p = 0.229; Fig 4B). In younger patients, the risk of major bleeding was lower than the risk of thrombotic events (hazard ratio 2.04, 95% confidence interval 1.24, 3.37, p = 0.0050), with notably few major bleeding events but a constant increase in thrombotic events during the 2^nd^ year. In contrast, no difference in the hazards for the two events could be shown in the elderly during the entire follow-up (hazard ratio 1.12, 95% confidence interval 0.74, 1.71, p = 0.593).

## Discussion

In this secondary analysis of a major stent trial, the following clinically important points can be highlighted: First, in patients undergoing stent implantation and treated with prasugrel-based DAPT, age appeared to be the most relevant predictor of major bleeding. While the risk of major bleeding increased with older age, the functional relationship with age was clearly non-linear with the lowest hazard at the age of 57 years and an exponential and significant increase only after 65 years. Of note, neither prasugrel dose nor patient weight emerged as a predictor of major bleeding. Second, in contrast to the non-linear relation between major bleeding and age, the association between thrombotic events and age was linear with a continuous increase in risk with older age. Third, the competing risk analysis showed that the risk balance was different between the two age groups: Younger patients i.e. patients <65 years of age, were at a higher risk for thrombotic events than for major bleeding throughout the entire 2 year trial period. In contrast, older patients, i.e. patients ≥65 years of age, were at a higher risk for major bleeding during the first year, while after the discontinuation of prasugrel the risks for both events approximated each other and the risk for thrombotic events became even more pronounced.

Major bleeding has been recognized as an important prognostic factor after PCI not only in ACS [8, 24] but also in an unselected PCI population [7]. Case fatality rates for major bleeding have been described in previous studies and are estimated to be over 12% [8, 24, 25]. Major bleeding is a frequent complication of DAPT and is more prevalent with newer compounds such as prasugrel and ticagrelor than with clopidogrel, most likely due to their higher and more consistent antiplatelet activity [5, 6]. The incidence of major bleeding after PCI has been described to be between 0.4% and 6.6% for different scenarios, with lower rates in stable patients and higher rates in patients with ACS [7–9, 15, 24, 26]. The association between bleeding and age has been recognized before with a higher risk in the elderly [9, 15, 27], but the non-linear relationship documented in the present analysis seems remarkable. However, different definitions for major bleeding have been used [28, 29] and make comparisons difficult. These definitions were developed for different settings, i.e., the more laboratory-based TIMI [28] and the predominantly clinic-based GUSTO definitions [29] for patients with acute myocardial infarction and thrombolysis and the more unifying BARC definitions specifically for patients undergoing PCI [18]. Thus, comparisons of bleeding rates between trials have to be done cautiously, considering the different definitions and patient situations [30].

The present competing risk analysis shows different risk patterns in patients younger and older than 65 years. While patients younger than 65 years had a bleeding rate that was lower than the associated thrombotic event rate during DAPT, the opposite was true for patients older than 65 years of age. During the 2^nd^ year of follow-up, i.e. after DAPT discontinuation, the bleeding rate no longer increased, but remained higher in older versus younger patients, whereas the rate of thrombotic events increased continuously and similarly in both age groups.

Therefore, two key questions in relation to the current use of DAPT in general and prasugrel in particular arise. First, our data suggest that the age-cut-off of 75 years for dose reduction of prasugrel in some previous studies [7, 15] was arbitrary and may have been too high. This cut-off age to reduce the daily prasugrel dose was selected based on TIMI-38 findings [6] and subsequently recommended by the European and U.S. authorities [11, 12]. However, the present analysis shows that the bleeding risk does not change with age in a linear fashion, but starts to increase exponentially already at 65 years of age, rather than at 75. This implies that dose reductions may already be necessary above age 65, questioning current recommendations. Second, DAPT in the elderly should be used with caution based on the increased bleeding risk, but at the same time might be necessary due to the similarly increased thrombotic risk, at least for a certain period of time. Overall, the continued increase in thrombotic risk argues for a prolonged duration of DAPT, which was tested in the DAPT trial [31]. It found a benefit of prolonged treatment with a further reduction of thrombotic events, but only a minority of patients were in the age group >65 years where the bleeding risk is markedly increased. Thus, patients older than 65 years of age pose a clinical dilemma, i.e., their exponentially increasing age-related bleeding risk may outweigh the benefit of reduced thrombotic events. In contrast, current guidelines recommend shorter DAPT durations for stable patients treated with DES, which may indeed reduce bleeding complications [32, 33]. This highlights the need for individualized treatment decisions based on available scores.

### Study limitations

This retrospective analysis has certain inherent limitations. Few patients with stable angina and treated with a BMS received only 1 month of DAPT, while some patients received prasugrel for stable angina. Guidelines at the time of the design of this trial advocated 12 month DAPT after DES implantation, while current guidelines support shorter DAPT durations in stable patients [33]. Based on the present data, the ideal duration of DAPT cannot be defined since only the competitive risks regarding bleeding and thrombotic events for 1 year DAPT were analyzed. Patients generally underwent PCI by femoral route in our study. The use of a radial access may lead to lower bleeding rates, but mainly with regard to BARC 1 and 2 bleedings (not reported here) and only for a short period of time after PCI. Ways to decrease the bleeding risk such as the use of the radial approach for PCI and a reduction of treatment time to 3 months in elective patients and to 6 months in patients treated for ACS receiving DES, as recommended by current guidelines [33] might be valuable options to further improve outcomes in high-risk patients undergoing PCI. A caveat remains in the continued increase of thrombotic events even after drug discontinuation as noted in the present analysis. Finally, we would like to emphasize that the analyses presented here are secondary analyses and thus of exploratory nature. Estimates, e.g. hazard ratios, should be interpreted taking the reported 95% confidence intervals into account. Although our results are strongly suggestive for changing risk patterns depending on patients’ age and thus question current recommendations for dose reductions, evidence from external validation is needed for confirmation.

## Conclusions

In patients undergoing stent implantation treated with prasugrel-based DAPT, age was the only independent predictor of major bleeding. While the risk of major bleeding increased with older age, the functional relationship was clearly non-linear with no significant change in the bleeding hazard up to the age of 57 years and an exponential and significant increase after 65 years. In contrast, there was a continuous age-related increase in the risk of thrombotic events in all patients up to 2 years. The competitive risk analysis showed that younger patients were at a higher risk for thrombotic events than for major bleeding. In contrast, older patients were at higher risk for major bleeding during the first year, while thereafter the risk curves for both events crossed indicating that the risk for thrombotic events became more predominant.

These findings imply that prasugrel standard dose may be appropriate up to 65 years of age. However, a dose reduction may be appropriate above 65 years but not only above 75 as recommended. The present competing risk analysis identifies an important clinical dilemma for daily practice, i.e., the continued increase in the risk of thrombotic events after the discontinuation of DAPT vs. the elevated bleeding risk on continued DAPT. Based on our data, the exponential increase in the risk of major bleeding may outweigh the benefit of prolonged DAPT at least in high-risk patients ≥65 years of age. Therefore, our data highlight the need for an individualized treatment approach in these patients.

## Supporting information

S1 STROBE Checklist (DOCX)

S1 R Code File Code for analysis. (R)

S1 Appendix Appendix 1 Authors and BASKET-PROVE II Investigators. (DOCX)

S1 Protocol Study protocol BASKET PROVE II. (PDF)

S1 Protocol Amendment (PDF)

## Abbreviations

ACS: acute coronary syndrome
BMS: bare metal stent
BARC: Bleeding Academic Research Consortium
DAPT: dual antiplatelet therapy
DES: drug eluting stent
PCI: percutaneous coronary intervention
BASKET PROVE II: Basel Kosten Effektivitäts Trial–Prospective Validation Examination II
GUSTO: Global Utilization of Streptokinase and Tissue Plasminogen Activator for Occluded Coronary Arteries
TRILOGY-ACS: Targeted Platelet Inhibition to Clarify the Optimal Strategy to Medically Manage Acute Coronary Syndromes
TRITON TIMI 38: Trial to Assess Improvement in Therapeutic Outcomes by Optimizing Platelet Inhibition with Prasugrel–Thrombolysis in Myocardial Infarction
