# Supplementary material for: Competing risks of major bleeding and thrombotic events with prasugrel-based dual antiplatelet therapy after stent implantation - An observational analysis from BASKET-PROVE II
Source: PLoS One. 2019 Jan 15;14(1):e0210821. doi: 10.1371/journal.pone.0210821 (PMC6333357; doi:10.1371/journal.pone.0210821)
Supplement: S1 Appendix — (DOCX) [file pone.0210821.s003.docx]

Appendix 1:

Authors and BASKET-PROVE II Investigators

*The Authors are as follows:*

Raban V. Jeger, M.D., Matthias Pfisterer, M.D., Søren Galatius, M.D., Ulrik Abildgaard, M.D., Christoph Naber, M.D., Hannes Alber, M.D., Franz Eberli, David J. Kurz, M.D., M.D., Giovanni Pedrazzini, M.D., André Vuilliomenet, M.D., Daniel Weilenmann, M.D., Hans Rickli, M.D., Stefanie von Felten, Deborah R. Vogt, Ph.D., Kim Wadt Hansen, M.D., Peter Rickenbacher, M.D., David Conen, M.D., Christian Müller, M.D., Stefan Osswald, M.D., Nicole Gilgen, M.D., Christoph Kaiser, M.D.,

*Additional authors/investigators:*

**University Hospital Basel, Switzerland:** Peter Buser, M.D., Christian Sticherling, M.D., Michael Zellweger, M.D., Patrick Hunziker, M.D., Michael Kuehne, M.D., Margarete Baumgartner, Andrea Harder-Allgöwer

**Gentofte University Hospital, Copenhagen, Denmark:**Jan Kyst Madsen M.D., Peter Riis-Hansen, M.D., Jan Skov Jensen, M.D., Anders Galloe, M.D., Kristian Wachtell, M.D., Rikke Sørensen M.D., Julie-Laage-Pedersen, Camilla Boehne, Susanne Rodholm, Lene Hogh Soderberg

**University Hospital Innsbruck, Austria:** Otmar Pachinger, M.D., Alois Süssenbacher, M.D., Maria Wanitschek, M.D., Nicolas Moes, M.D., Matthias Frick, M.D., Florian Hintringer, M.D., Guy Joseph Friedrich, M.D., Gerhard Pölzl, M.D., Bernhard Metzler, M.D., Thomas Bartel, M.D., Katja Heitmair-Wietzorrek, Jakob Dörler

**Elisabeth-Krankenhaus Essen, Germany:** Thomas Schnitz, M.D., Werner Nickl, M.D., Walter Richter, M.D., Neyhbi Ljumani, M.D., Georg V. Sabin, M.D., Bernhard Grosch, M.D., Christoph Wald, M.D., Günther Szurawitzki, M.D., Dietmar Schmitz, M.D., Oliver Bruder, M.D., Holger-Carsten Eberle, M.D., Ingo Voigt, M.D., Heribert Pütz-Hellweg, M.D., Alexander Wolf, M.D., Karsten Meuter, M.D., Vanessa Reuter, Elisabeth Blank

**Cardiocentro Lugano, Switzerland:** Tiziano Moccetti, M.D., Mariagrazia Rossi, M.D., Iveta Petrova Slater, M.D., Angelo Auricchio, M.D., Augusto F. Gallino, M.D., Elena Pasotti, M.D., Carmela Crljenica, M.D., Monya Bondino, M.D., Fulvio Bomio-Pacciorini, Alessandro G.A. Del Buffalo, M.D., Daniel Sürder, M.D., Cristina Monti Panzeri, Christa Camporini, Elena Giorgetta, Andrea Rocco Belissimo, Lorena Chiumiento, Grazia Dolci-Pasotti, Adriana Anesini, Simona Maspoli, Manuela Mombelli, Simona Polledri, Giada Poni

**State Hospital St. Gallen, Switzerland:** Lukas Trachsel M.D., Dominique Nüssli, M.D., Peter Ammann, M.D., Philipp Haager, M.D., Lucas Joerg, M.D., Micha Maeder, M.D., Hans Roelli, M.D., Franziska Rohner, M.D., Michaela Gemperle, Getrude Steven, Kathrin Manusco, Mirjam Schefer

**Triemlispital Zürich, Switzerland:** David Tüller, M.D., Ivano Reho, M.D., Alain M. Bernheim, M.D., Rainer Zbinden, M.D., Christina Deml, M.D., Ulrike Dümmel, M.D., Claudia Liedtke, Claudia Frey, Franziska Gerig

**State Hospital Aarau, Switzerland:** Martin Steiner, M.D., Igal Moarof, M.D., Jens G Hellige, M.D., Sandra Popp.

*The affiliations of the authors are as follows:* University Hospital, Basel; State Hospital, Aarau; Triemli Hospital, Zurich; State Hospital, St. Gallen; Cardiocentro, Lugano - all in Switzerland; Gentofte University Hospital, Copenhagen, Denmark; University Hospital, Innsbruck, Austria; Elisabeth-Krankenhaus, Essen, Germany

*The BASKET-PROVE II Investigators:*

**Co-Principal Investigators:** Christoph Kaiser, M.D., University Hospital, Basel, Switzerland; Matthias Pfisterer, M.D., University Hospital, Basel, Switzerland

**Steering Committee:** Matthias Pfisterer, M.D., Basel, Switzerland; Christoph Kaiser, M.D., Basel Switzerland; Raban Jeger, M.D., Switzerland; Søren Galatius, M.D., Copenhagen, Denmark; Franz Eberli, M.D., Zürich, Switzerland; Hannes Alber, M.D., Innsbruck, Austria; Hans Rickli, M.D., St. Gallen, Switzerland; Giovanni Pedrazzini, M.D., Lugano, Switzerland; André Vuillomenet, M.D., Aarau, Switzerland; Christoph Naber, M.D., Essen, Germany

**Participating centers (number of included patients in parenthesis):** University Hospital, Basel, Switzerland (398); Gentofte University Hospital, Copenhagen, Denmark (522); University Hospital, Innsbruck, Austria (433); Elisabeth-Krankenhaus, Essen, Germany (331); Triemlispital, Zürich, Switzerland (229); State Hospital, St. Gallen, Switzerland (114); Cardiocentro, Lugano, Switzerland (160); State Hospital, Aarau, Switzerland (104)

**Critical Events Committee:** Peter Rickenbacher, M.D.; Cardiology, Bruderholz State Hospital, Switzerland (chair); Christian Müller, M.D.; University Hospital, Basel, Switzerland; David Conen, M.D., University Hospital, Basel, Switzerland

**Data and Monitoring Center:** Nicole Gilgen, M.D., Margarethe Baumartner, Andrea Harder-Allgöwer, University Hospital, Basel, Switzerland; Simon. Spitzmüller, M.D. State Hospital Baden, Switzerland

**Statistical Center:** Stefanie von Felten, Ph.D., Deborah R. Vogt, Ph.D., Kim Wadt Hansen, M.D., Pascal Benkert, Ph.D., Clinical Trial Unit, University Hospital, Basel, Switzerland
