# Supplementary material for: Competing risks of major bleeding and thrombotic events with prasugrel-based dual antiplatelet therapy after stent implantation - An observational analysis from BASKET-PROVE II
Source: PLoS One. 2019 Jan 15;14(1):e0210821. doi: 10.1371/journal.pone.0210821 (PMC6333357; doi:10.1371/journal.pone.0210821)
Supplement: S1 Protocol — (PDF) [file pone.0210821.s004.pdf]

Protocol Title

Evaluation of late clinical events after drug-eluting versus bare-metal stents in patients at risk:

BAsel Stent Kosten Effektivitäts Trial – PROspective Validation Examination Part II

BASKET-PROVE II

(November 09, 2009)

Sponsor: University Hospital, Department Cardiology

Principal Investigator: PD Dr. med. Christoph Kaiser

Signature:________________________________

Table of contents

Page 3: Protocol Synopsis

Page 5: Background

Page 9: Aims

Page 11: Patients (Inclusion/Exclusion)

Page 12: Study Design

Page 14: Follow-Up

Page 14: Concomitant Therapy

Page 15: Treatment of In-Stent-Restenosis

Page 16: Endpoint Definition

Page 17: Sample Size Calculation

Page 18: Data Handling Analyses

Page 19: Finances/ Insurance

Page 20: Ethics / Time Plan

Page 21: Study Organisation

Page 22: Clinical Relevance

Page 23: Appendix 1 (FDA Statement on Coronary Drug-Eluting Stents)

Page 25 Apendix 2 (Pharmacovigilance)

Page 27: References

Page 33: Abbreviations

PROTOCOL SYNOPSIS

Evaluation of late clinical events after drug-eluting versus bare-metal stents in patients at risk:
BAsel Stent Kosten Effektivitäts Trial – PROspective Validation Examination, Part II
(BASKET-PROVE II)

Background:
Retrospective detailed analyses of long-term BASKET findings identified patients with large DES ($\geq$3mm Stents) as patients at risk for late cardiac death/nonfatal MI. To prove this findings, BP compared 2-year cardiac death/MI rates in patients treated with a 2nd generation BMS (Vision®) versus standard DES (Cypher®) and versus a 2nd generation DES (XienceV®). Results of 2324 included patients with 12 months aspirin/clopidogrel treatment will be available in 2010. In view of new DES with absorbable polymers and new BMS with thin struts and biocompatible polymers, BP-II will be launched to test their comparative clinical safety up to 5 years if treated with an aspirin/prasugrel combination, since prasugrel halved stent thrombosis rates compared to clopidogrel in a large ACS trial.

Aims :
- Primary aim: To compare 24 month safety (cardiac death/MI rates) of a new tpye BMS (P with thin cobalt-chromium struts and biocompatible polymer) in a 1:2 randomisation to two new types of DES (X: durable polymer, low dose –limus drug; N:biodegradable polymer with –limus drug) in unselected patients at low risk for restenosis (>3mm stents only) on the background of contemporary antiplatelet therapy.
- Secondray aims:
  - a) To compare the efficacy of BMS vs. DES and the combined endpoint of safety and efficacy of the primary comparison (BMS vs. DES)
  - b) To compare safety and efficacy of each of the new DES to that of the BMS, i.e. X vs P and N vs P
  - c) To compare safety and efficacy of the two new DES, i.e. X vs. N
  - d) & e) Historical comparisons with BP : early BMS strategy (Vision® stent with 12 months Clopidogrel) with the new BMS strategy (P with 1 month Prasugrel in elective and 12 months in ACS-patients); Safety and efficacy of the the X-stent in similar patient groups receiving an antiplatelet therapy of 12 months Clopidogrel (BP) vs. those receiving 12 month Prasugrel (BPII)

Set-up:
Multicenter prospective randomized trial.

Patient inclusion:
Unselected series of patients in need of large ($\geq$3mm) stents only in native vessels irrespective of clinical indication.

Patient exclusion:
In-stent restenosis, Left-main disease, cardiogenic shock, planned surgery <12months, increased bleeding risk, no compliance expected, History of stroke or TIA.

Randomization:
By centre using sealed envelopes 1:2 for BMS to DES and 1:1: for the DES subgroups.

Follow-up:
After 2,3 and 5 years by structured questionnaire.

Statistics:
A sample size of 800 patients/stent type (753 plus compensation for a withdrawal rate of 5 to 6%) would provide a power of more than 90% to detect, at an alpha level of 0.05 in a two

tailed test, a significant difference between the BMS group and the DES group. This sample size will also allow to detect a significant difference between the BMS and each of the DES groups wit a power of 80% at an alpha level of 0.027.
Intention to treat analysis: bivariable group comparisons will be first performed, then time to event will be evaluated both by Kaplan Meier statistics and Cox-regression analysis taking into account influential factors.

Endpoints:
- 1° end-point = freedom of combination of cardiac death (all death not clearly of extra cardiac origin and documented non-fatal MI after 24 months.
- 2° end-points = non-MI related TVR; MACE = 1° end -point events + non-MI related TVR ;1° end-point events up to 18 months (for comparison with BASKET and BASKET-LATE); stent thrombosis according to the ARC-definitions; components of the 1° end-points; non-cardiac death (total death); major non-CABG bleeding (need for surgery, blood transfusions, cerebral haemorrhages) during dual antiplatelet therapy (up to twelve months); “net clinical benefit” = 1° end-point + bleeding; long-term follow-up after 36 and 60 months; subgroups with diabetes, acute coronary syndrome, ST-elevation MI, need for GP IIb/IIIa inhibitors, lesions >25 mm.
- End-points will be adjudicated by an independent Critical Events Committee (CEC) blinded to the stent type used.

Clinical Relevance:
If findings of the retrospective analysis of BASKET/BASKET-LATE are verified by the prospective BASKET-PROVE and BASKET-PROVE II trials, then 1st generation DES may no longer be justified in large native vessel stenting. This would reduce the late harm which includes late cardiac death, which is most relevant in view of the >6 million DES implanted per year worldwide today. In addition, it would reduce costs considerably due to the much lower price of BMS vs. DES. If, however, findings do not confirm results of BASKET-LATE then the “late harm” observed there was a “chance” finding and no longer relevant, or to a much lesser degree than postulated in BASKET-LATE. If findings differ between different stent types the results will help define the most safe and effective stent type to treat patients with the need for large vessel stenting. In addition, results of the study will help to understand the mechanisms of late stent thrombosis: if late clinical events related to late stent thrombosis do no longer or to a lesser extent occur with newest generation DES with a bioabsorbable polymer, then late stent thrombosis is most likely due to the polymer and not to the drug or drug-dose used. By the historical comparison of the XienceV® stent-arms of BASKET-PROVEII with BASKET-PROVE, the analysis will provide also the unique chance to test the performance of an up-to-date DES with a dual antiplatelet therapy with Prasugrel versus Clopidogrel in an unselected real-world population. – Thus, these findings should have major impact on the current use of coronary stents and our understanding of possible reasons for late stent thrombosis.

Abbreviations:
BP = BASKET-PROVE ; BP-II = BASKET-PROVE II
X = XienceV®= 2nd generation DES with lower “limus”-drug dose.
N = Nobori® = DES with bioabsorbable polymer and a novel “limus”-drug (biolimus)
P = PROKinetik Energy® = BMS with thin struts and a biocompatible polymer

STUDY PROTOCOL

1. Background

BASKET was a prospective study to test the cost-effectiveness of drug-eluting stents (DES) versus bare-metal stents (BMS) in a „real-world“ setting, i.e. in all-comers for percutaneous coronary interventions (PCI) irrespective of indication (1). This trial showed that DES (Cypher®, Cordis, Johnson & Johnson, Miami Lakes, Florida, USA or TAXUS®, Boston Scientific Corporation, Natic, Massachusetts, USA, randomized 1:1) were not cost-effective compared to BMS (Vision®, Guidant Corporation, Indiannapolis, Indiana, USA, DES:BMS = 2:1 randomization) at an incremental cost-effectiveness ratio to prevent one clinical event of € 18’031 after 6 months or € 53’094 after 18 months (2). In high risk subgroups such as small vessel stenting, multivessel disease, long lesions, multiple stenting and elderly patients, however, DES were more cost-effective or even cost-saving. BASKET-LAte Thrombotic Events (BASKET-LATE), a follow-up investigation of clinical events up to 18 months (month 7 to 18) after discontinuation of clopidogrel identified an excess in late cardiac death/non-fatal myocardial infarction (MI) in patients treated with DES compared to those treated with BMS (3), which was true also after 36 months (4). There was a strong indication that these late clinical events were related to late stent thrombosis. These findings were indirectly confirmed by a large registry of patients from Berne and Rotterdam (5) showing a rate of late angiographically documented stent thrombosis of 0,6% per year during the first 3 years of follow-up in patients treated with DES, an effect not seen after BMS stenting.

Predictors of late clinical events in BASKET-LATE were use of glycoprotein IIb/IIIa inhibitors (as measure of acute coronary syndromes or suboptimal PCI results), prior MI and DES use, and in the Berne/Rotterdam registry the only independent predictor of late stent thrombosis was acute coronary syndrome. A more profound analysis of subgroups benefiting from DES was performed based on the overall BASKET/BASKET-LATE data after 18 months (6).

It suggested that patients with small vessel (<3,0 mm) and bypass graft stenting had a highly significant benefit of DES, not only in reduction of target vessel revascularization (TVR), but also in a reduced rate of cardiac death/non-fatal MI. In contrast, data suggested that patients with large native vessel stenting do not seem to benefit from DES and may even have late harm (see Fig.). These findings were confirmed after 36 months (4) and are in accordance with earlier retrospective analyses from different trials showing a particular benefit of DES in small vessels or long lesions (7-11), diabetes mellitus (12-14) and bypass graft stenting (15,16).

In order to prove this hypothesis, BASKET-PROVE (Evaluation of late clinical events after drug-eluting versus bare-metal stents in patients at risk: BAsel Stent Kosten Effektivitäts Trial – PROspective Validation Examination) was initiated in 11 centres in Switzerland, Austria, Denmark and Italy in 2007 (17). In this trial a total of 2324 patients in need for large (>3mm stents only) native vessel stenting only were randomised 1:1:1 to either treatment with Cypher-Select® (standard 1st generation DES) versus Vision® (3rd generation cobalt-chromium BMS) versus XienceV® (DES with a lower dose of a "limus" drug, i.e.Everolimus). The results of the primary endpoint (freedom of combination of cardiac death and non-fatal-MI) will be available by summer 2010.

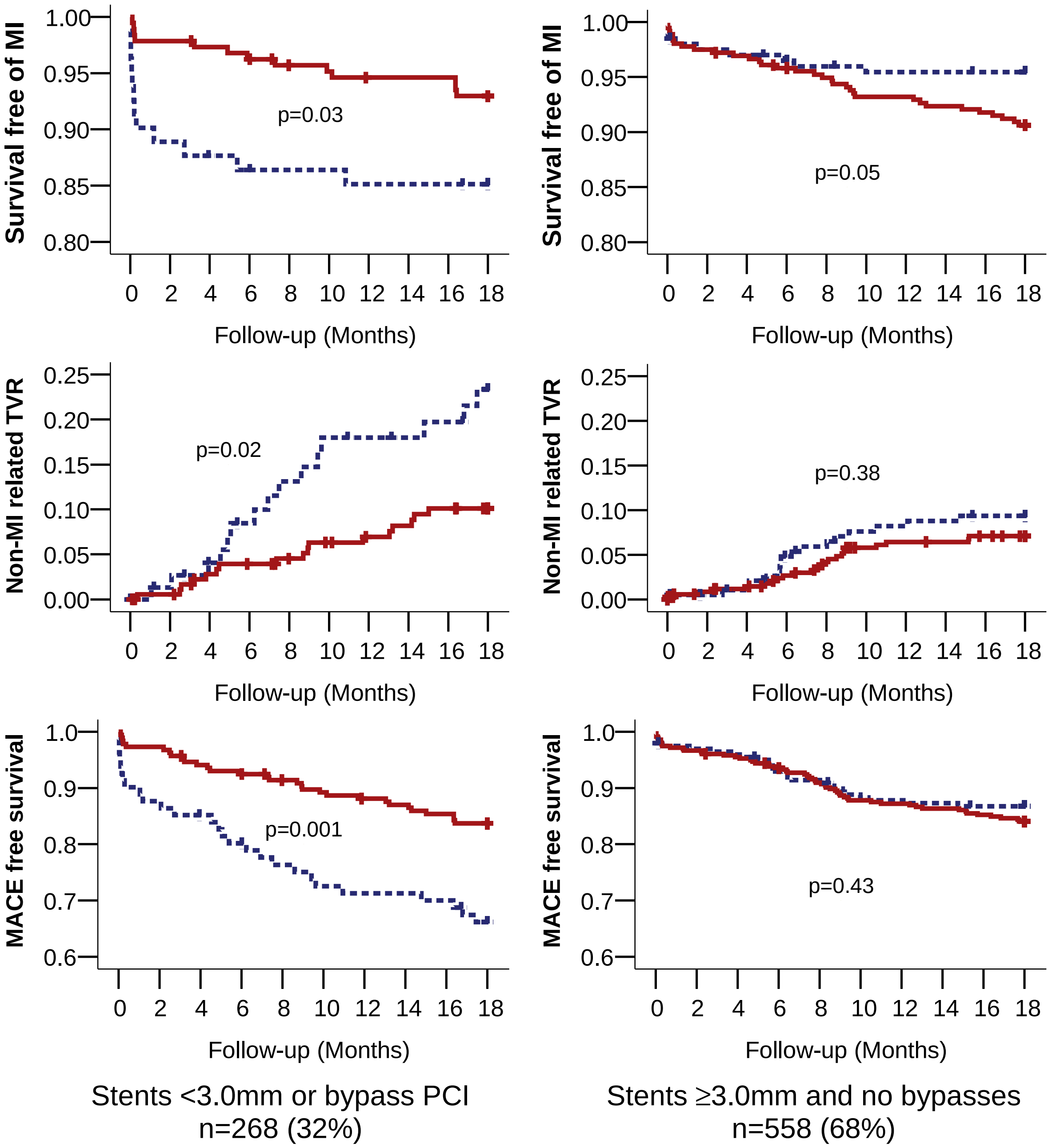

Comparison of bare-metal stents (BMS; blue dotted line) and drug-eluting stents (DES: red continuous line) regarding cardiac survival free of non-fatal myocardial infarction (MI;top), cumulative rate of non-infarct related target vessel revascularisation (TVR; middle), and survival free of major adverse cardiac events (MACE; bottom) in patients treated with small stents <3mm only or with PCI in bypass grafts (left side) and in patients treated with large stents ≥3mm in native vessels (right side).

Reasons for late stent thromboses lay in delayed endothelial healing and hypersensitivity reactions as noted in autopsy (18) and angioscopic (19) studies and it has been speculated whether prolonged dual antiplatelet therapy (Aspirin and Clopidogrel) may prevent these events. Since no clustering of events after clopidogrel discontinuation was noted in BASKET-LATE (3) nor in the Berne/Rotterdam registry (5) and since events occurred on dual (1/4), mono (1/2) or no (1/4) antiplatelet therapy, prolonged dual antiplatelet therapy may not be the definite solution of the problem. However, based on registry data suggesting a marked benefit of prolonged dual antiplatelet therapy in patients with ACS, clopidogrel was recommended for 9-12 months in these patients (20,21). In view of the fact that the main drug effects are observed within the initial 4 weeks after implantation, prolonged duration of drug action of the DES seems not be the reason for these late events. Therefore, the polymer, "carrier" of the drug on the stent which remains on the surface of the stents after total drug release or the initial drug dose may be relevant factors for delayed healing. As a consequence, DES with biodegradable polymers have been introduced with promising results (22). Simultaneously, the effectiveness of BMS could be improved by further reducing strut thickness (23) and introducing biocompatible coatings (24).

Currently prolonged antiplatelet therapy with Aspirin and Clopidogrel is currently recommended by the FDA and ESC-Guidelines for 12 months after DES (Appendix 1). Prasugrel, a novel thienopyridine, has been investigated in patients with ACS and coronary stenting (25). Compared to standard Clopidogrel therapy early and late stent thrombosis could be reduced by approximatively 50% in DES and BMS treated patients. Prasugrel will be on the market in Switzerland and the EU by Summer 2009 and may rapidly replace Clopidogrel in all stent procedures.

2. AIMS

These recent findings and analyses are the basis for two relevant questions which will be addressed prospectively in BASKET-PROVE II.

The primary aim will be:

Tto assess and compare the 24 month safety (cardiac death/MI) of a new type of BMS with new types of DES in patients at low risk of restenosis, i.e. receiving stents of ≥3,0mm diameter only, on the background of contemporary antiplatelet therapy. The new BMS, with thin cobalt-chromium struts and a biocompatible coating (Prokinetik®), will be compared (in a 1:2 randomisation) to two new DES:

- the most widely used 2nd generation DES with a durable polymer and a low dose -limus drug (XienceV®), which was already used in BASKET-PROVE),
- a DES with a biodegradable polymer and a -limus drug (biolimus) (Nobori®))

The antiplatelet regime will be Aspirin 100mg and Prasugrel 10mg daily for all patients. Based on current recommendations, this will be given for 1 month after BMS in stable patients and for 12 months after DES implantation and after BMS implantation in patients with ACS.

The secondary aims will be:

A. To compare the efficacy of BMS vs. DES and then the combined endpoint of safety and efficacy of the primary comparison (BMS vs. DES)

B. To compare safety and efficacy of each of the new DES to that of the BMS, i.e. XienceV® vs Prokinetik® and Nobori® vs Prokinetik® (both, randomized 1:1)

C. To compare safety and efficacy of the two new DES, i.e. XienceV® vs. Nobori®

There will be also the possibility to compare findings from BASKET-PROVE II with those of the predecessor study BASKET-PROVE. This will have the following additional secondary aims:

D. To compare safety and efficacy of the earlier BMS strategy (Vision® stent with 12 month clopidogrel), as a historical control, with the new BMS strategy (Prokinetik®) stent with 1 month Prasugrel in elective patients and 12 months in patients with ACS)

E. To compare safety and efficacy of the the XienceV® stent in similar patient groups (same indications/exclusions, similar sample size) receiving an antiplatelet therapy of 12 month Clopidogrel (BASKET-PROVE) vs. those receiving 12 month Prasugrel (BASKET-PROVE II) in a "historical" control analysis, evaluating also bleeding rates and "net clinical benefit"

3. <u>Patients</u>

Included will be:

- all comers, 24 hours a day, 7 days a week, irrespective of indication for PCI
- in need of large (≥3.0 mm stents only) native vessel stenting

Excluded will be patients with

- in-Stent Restenosis or in-Stent Thrombosis
- bypass graft disease to be stented
- main stem disease to be stented
- cardiogenic shock by clinical assessment (signs of organ hypoperfusion)
- planned surgery within the next 12 months
- oral anticoagulation needed (artificial heart valves, atrial fibrillation) or chronic haemorrhagic diathesis
- active bleeding disorders
- index-PCI = planned PCI of additional lesion
- no FU expected/possible
- History of stroke or TIA (contrindication for prasugrel)
- known severe hypersensitivity reaction to ASS and/or Prasugrel
- no compliance expected / no informed consent given
- enrolled in another study

4. Study design

BASKET-PROVE II will be a prospective randomized open-label multicenter trial Randomization will be 2:1 for DES to BMS. The DES group will be sub-randomized 1:1 for XienceV® to Nobori®.

All patients will receive a loading dose of Aspirin (300-500mg) and of Prasugrel (60mg) initially, followed by a maintenance dose of Aspirin 100mg and Prasugrel 10mg daily for 1 month after BMS in elective patients and 12 month after DES implantation and after BMS implantation in patients with ACS as currently recommended. All other treatments will be given according to current guidelines and patient needs (based on local treating physicians prescriptions, see point 6)

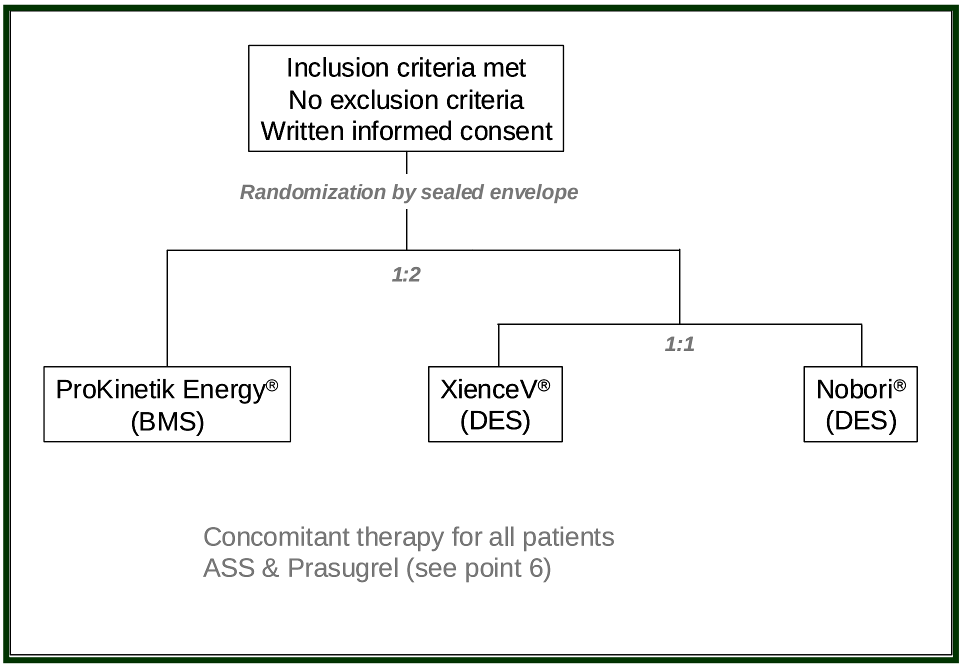

Patients without emergency intervention will be included in the study after giving written informed consent. Since BASKET-PROVE II is an all-comer, real-world trial, the quality of the trial strongly depends on inclusion of not only elective, but also emergency patients with acute coronary syndrome and myocardial infarction. Since

these patients commonly suffer from acute chest pain and dyspnea and since every minute of time delay would adversely affect prognosis, it is ethically not acceptable to postpone the intervention to obtain written informed consent prior to study inclusion (in accordance with the Swiss federal law on drugs and medical devices [Heilmittelgesetz], Art. 56). Thus, as soon as coronary intervention is planned and the patient fulfils study entry criteria, the patient will be informed about the trial and asked for oral consent by the operator in charge. This will be done in parallel to preparation of material for coronary intervention, guaranteeing no time delay by inclusion of the patient in the study. Oral consent will be documented on the informed consent form by a second medical person not being involved in the trial. Only with oral consent, the patient will be included in the study and will have to give the definitive written informed consent after the PCI-procedure. Baseline PCI consists of the index procedure and of all planned PCI thereafter (“two step”).

5. Follow-up

- Follow-up will be clinical with no “routine” follow-up angiography allowed (follow-up angiography only for relevant new symptoms!)

  After 24 , 36 and 60 months a structured questionnaire will be sent to all study patients regarding clinical events (1st, 2nd endpoints) as well as drugs used. In addition, quality of life will be assessed using the self-admistered EQ-5D questionnaire, which consists of a self classifier and and a visual analogue scale, at baseline and after 24, 36 and 60 months (26,27). All events will be documented based on hospital and/or private physician charts/discharge letters.

- A separate registry will be conducted including all patients treated with PCI during the time of randomisation but not included in the main trial.

6. Concomitant Therapy

Concomitant therapy will be

- Aspirin * 100 mg/day throughout
- statin throughout
- Prasugrel * :

  Dosage: -10 mg/day in patients ≤75 years and body weight ≥60kg

  - 5 mg/day in patients >75 years or body weight <60kg

  Duration: - 12 months after DES or after BMS in patients with ACS

- 1 month after BMS in elective cases

Loading dose: - 60mg

If loaded with Clopidogrel: - additional loading with Prasugrel **

- other treatments as clinically indicated

(*after appropriate loading dosages if indicated)

(**: Reasons for additional loading dose with Prasugrel:
- Same and only mode of action for both drugs at the site of the platelet (irreversible block of P2Y12 - ADP-receptor
- Concentration of active metabolite after loading dose 12 times lower with Clopidogrel compared to Prasugrel
- If no additional loading dose: different levels of platelet inhibition for patients with Clopidogrel and Prasugrel in the early phase
- With additional loading dose: same level of platelet inhibition for all patients in the early phase)

7. Treatment of In-Stent-Restenosis:

In-Stent Restenosis in XienceV®: Treatment with Nobori®

In-Stent-Restesnosis in Nobori®: Treatment with XienceV®

In-Stent Restenosis in PROKinetic Energy®: Treatment with XienceV®

8. End-point definitions

1° end-point = freedom of combination of

- cardiac death (all death not clearly of extra cardiac origin)
- documented non-fatal MI (according to the current ESC-guidelines (28))

after 24 months.

2° end-points =

- non-MI related TVR
- major adverse cardiac events (MACE) = 1° end-point events + non-MI related TVR
- 1° end-point events up to 18 months (for comparison with BASKET and BASKET-LATE)
- stent thrombosis according to the ARC-definitions (29)
- components of the 1° end-points
- non-cardiac death, total death
- major non-CABG bleeding (need for surgery, blood transfusions, cerebral haemorrhages) during dual antiplatelet therapy (up to twelve months) – "net clinical benefit" = 1° end-point + bleeding.
- long-term follow-up after 36 and 60 months
- subgroups with
  - diabetes
  - acute coronary syndrome
  - ST-elevation MI
  - need for GP IIb/IIIa inhibitors
  - lesions >25 mm

End-points will be adjudicated by an independent Critical Events Committee (CEC) blinded to the stent type used.

9. Sample Size calculation

In the 558 patients of BASKET/BASKET-LATE with treatment of large native vessels, cardiac deaths and non-fatal MIs were seen in 6% of BMS compared to 11.2% of DES treated patients (p=0.05) after a follow-up of 18 months. Based on a slightly more conservative assumption (i.e. 6.0 vs. 10.5% after follow-up of 24 months) and on the design of the proposed study, a total of 753 patients for the BMS group and a total of 1506 patients for the DES group (which includes the XienceV® and Nobori® groups) would have to be included to reject the null hypothesis that the event rates for the DES and BMS groups are equal at an alpha level=0.05 in a two-tailed test with probability of more than 90% (power). In view of an expected withdrawal rate of 5-6% and the 2:1 randomization, we plan to enroll 3x800 patients into the study or a total of 2400 patients; i.e. we could still detect a relative difference of 22% between BMS and DES at an alpha level of .027 with a power of 70% if the BMS rate stays the same, and with a slighter higher power an an alpha level of .05 if the BMS rate would be 5.5% instead of 6%.To address the second aim (comparison of BMS with each of the two DES) within BASKET-PROVE II, the sample size of 800 patients per stent group will allow to reject the null hypotheses that the event rates for each of the DES and the BMS are equal at an alpha level of 0.027 in a two-tailed test with a power of 80%. A simulation showed that a nominal alpha of 0.027 is appropriate to adjust for the duplicity of testing in the following setting: if three groups of equal size are compared with respect to the relative frequency of a certain outcome, the z-standardized differences (p2-p1) and (p3-p1) follow a 2-dimensional normal

distribution with variance 1 and covariance 0.5 under the null hypothesis. The Fisher's exact test will be used to evaluate the above hypotheses.

If we assume a total inclusion rate of 170 patients per month as in BASKET-PROVE by all participating centres, we should finish inclusion within 13-15 months.If results of BASKET-PROVE should differ markedly from these assumptions, i.e. difference in rates in cardiac death/MI of BMS patients vs. XienceV® patients <3.0% or >5.0%, then these sample size considerations will have to be reassessed .

10. Data handling and Analyses

Data management will be based on experience in other large multicenter trials (17,30). Thus, an internet-based electronic CRF will be used for data management. A secure website, accessible by password only, will be provided for data entry. Data will be stored centrally using an SQL-database. Quality of data will be assured by regular on-site monitoring visits, comparing data entered in CRFs with source documents. After monitoring, changes in CRF will be done electronically and only be possible by monitors and study coordination.

The following data will be recorded, in analogy to BASKET-PROVE I:

- Patient history and demographics
- CAD risk factors
- Comorbidities
- Indication for PCI
- Baseline angiographic and procedural characteristics
- Relevant data of stent use and concomitant drug use
- Serious adverse events during follow-up

- Events during follow-up (i.e. all hospitalisations, all coronary interventions, all bleeding, death)
- EQ-5D

For the statisitcal analysis, the data will be extracted from this database and transferred to and analysed in the commercially available statistical package SPSS v.15.0.

Bivariable comparisons of the DES group with the BMS group will be done using Fisher's exact test for proportions and the Mann-Whitney U-test, for continuous variables. P-values for the comparisons of each of the DES groups to the BMS group will be adjusted using the Bonferroni-Holm method if multiple comparisons of proportions or the Dunnett-Hsu if multiple comparisons of survival curves are performed. Time to event will be evaluated both by Kaplan Meier statistics and multivariable Cox-regression analysis taking into account further influential variables.

Historical comparisons of net clinical benefits will be only of descriptive character.

A priori, missing values will not be imputed. Losses to follow-up will be incorporated since survival analysis will be performed. If outliers values are observed, each will be examined. If it is thought that their cause is of clinical nature, they would be kept in the analysis, otherwise they will be set to missing.

11. <u>Finances/Insurance</u>

The stents will be used as clinically indicated and therefore paid by the health insurance. In fact, the rate of the more expensive DES will only be 2/3 in this study,

compared to the average of 85% overall in Switzerland 2007, resulting in reduced overall health care costs.

Follow-up questionnaires and event documentation as well as analyses will be paid by a special study grant.

The study will be planned, conducted, analysed and interpreted entirely independently from industry and will be entirely founded ba research grants of the Basel Cardiovascular Research Foundation and the University of Basel. The concomitant medication is provided for free to all patients by the European Alliance of Ely Lilly Europe.

Since no “investigational” devices will be used (all stents are CE marked and freely available on the market in Switzerland and Denmark) this is in fact an observational clinical study only. A special insurance for all included patients in Switzerland, Asutria and Germany will be effected with the Zürich insurance-company before enrolment of the first patient.

12. Ethics

The study protocol undergoes approval by the Ethics Committee of each centre. Each patient will be asked to give written informed consent to participate in this study and its follow-up (see above). The study will be conducted according to the GCP-Guidelines.

13. Time plan

Approval of the protocol: Summer 2009

Ethical approval: Autumn 2009

Start of study: January 2010 (Basel; other centres as soon as ethical approval is available at each centre)

Inclusion completed: Spring 2011

Last follow-up completed: Spring 2013 (24months)
Spring 2015 (60 months)

First presentation of results: ESC 2013

14. Study organisation:

Participating centres and investigators will be the following:

- **Basel, University Hospital:** R. Jeger (Investigator), M. Handke (Co-Investigator), H.-P. Brunner, P. Buser
- **Lugano, Cardiocentro:** G. Pedrazzini (Investigator),T Moccetti
- **St. Gallen:** H. Rickli (Investigator), D. Weilenmann
- **Zürich, Stadtspital Triemli**: F. Eberli (Investigator), D. Kurz
- **Aarau, Kantonsspital**: A. Vuilliomenet (Investigator)
- **Copenhagen, Denmark, Gentofte University Hospital**: S. Galatius (Investigator), JK.Madsen, JS Jensen
- **Innsbruck, University Hospital**: O. Pachinger (Investigator), H. Alber
- **Essen, Germany, Elisabeth Krankenhaus :** PD Dr. C. Naber (Investigator)

Principal Investigator: C. Kaiser, Basel

Principal Co-Investigator: M. Pfisterer, Basel

Steering Committee:

C. Kaiser (chair), M. Pfisterer, R. Jeger, O. Pachinger, G. Pedrazzini, H. Rickli, F. Eberli, S. Galatius, A. Vuilliomenet, C. Naber

Data Centre/Statistics: H.-P. Brunner, University of Maastricht, The Netherlands; R. Jeger, University Hospital Basel, Switzerland

Critical Events Committee: P. Rickenbacher (chair), University Hsopital Bruderholz, Switzerland; D. Conen and C. Müller, Department of Internal Medicine, University Hospital Basel, Switzerland; P. Hunziker, Deaprtment of Intensive Care Medicine. University Hospital Basel, Switzerland

15. Clinical Relevance

If findings of the retrospective analysis of BASKET/BASKET-LATE (12) are verified by the prospective BASKET-PROVE and BASKET-PROVE II trials, then the 1st generation DES may no longer be used for large native vessel stenting. This would reduce the late harm related to late stent thrombosis which includes late cardiac death, which is most relevant in view of the >6 million DES implanted per year worldwide today. In addition, it would reduce costs considerably due to the much lower price of BMS vs. DES. If, however, findings do not confirm results of BASKET-LATE then the “late harm” observed there was a “chance” finding and not relevant further, or to a much lesser degree than postulated in BASKET-LATE. If findings differ between different stent types, results will help define the most safe and effective stent type to treat patients in need for large vessel stenting. In addition, results of the study will help to understand mechanisms of late stent thrombosis: if

late clinical events related to late stent thrombosis do no longer or to a lesser extent occur with newest generation DES with a bioabsorbable polymer, then late stent thrombosis is most likely due to the polymer and not to the drug or drug-dose used. By the historical comparison of the XienceV® stent-arms of BASKET-PROVEII with BASKET-PROVE, the analysis will provide also the unique chance to test the performance of the most widely used DES with dual antiplatelet therapy based on Prasugrel versus Clopidogrel in an unselected real-world population. – Thus, these findings should have major impact on the current use of coronary stents and our understanding of possible reasons for late stent thrombosis.

Appendix 1

SEPT 14, 2006

FDA Statement on Coronary Drug-Eluting Stents

FDA is providing the following information in response to inquiries asking for the agency's position on adverse events related to coronary drug-eluting stents (DES). This information describes our position at this time and does not represent new agency policy.

FDA has been closely monitoring DES since they came to the United States market in 2003 and 2004 - and will continue to do so.

We are aware of recent data suggesting a small but significant increase in the rate of death and myocardial infarction (heart attack) possibly due to stent thrombosis (a blood clot in the stent) in patients treated with DES. The specific studies that have prompted recent media inquiries are the BASKET-LATE study (presented at the March 2006 American College of Cardiology Scientific Sessions in Atlanta, Ga.) and more recently, the Camenzind meta-analysis (presented at the September 2006 European Society of Cardiology Annual Meeting/World Congress of Cardiology Meeting in Barcelona, Spain). The small but significant increase in the rate of death and myocardial infarction observed in these studies was noted in patients followed 18 months to 3 years after stent implantation.

While the studies presented at the Atlanta and Barcelona meetings have raised important questions, the data we currently have do not allow us to fully characterize the mechanism, risks, and incidence of DES thrombosis. A more formal evaluation of the data in these studies is necessary, and any conclusions are dependent upon a thorough peer review. FDA intends to more formally evaluate the studies presented in Atlanta and Barcelona.

Stent thrombosis in patients who receive DES is a primary area of interest for the agency because of the potential for serious adverse outcomes-even though stent thrombosis occurs at low rates. Over the past two months, the agency has met with both manufacturers of the FDA-approved approved DES to discuss any information and perspectives they have that may be pertinent to this issue. In assessing the risk of stent thrombosis, we remain keenly interested in the long-term follow-up of patients enrolled in the original pivotal DES randomized trials as well as those in the more complex patient and lesion subsets (for example, patients with diabetes; acute myocardial infarction or multiple vessel disease; or lesions involving arterial bifurcations, the left main coronary artery, and long arterial segments) who are currently being treated in "real world" randomized and registry studies.

FDA also continues to closely evaluate information related to the duration of treatment with clopidogrel (Plavix), a drug used in combination with aspirin to reduce/prevent clotting in DES patients. Although the duration of clopidogrel appeared to be adequate for the selected patients in the original clinical trials conducted to support FDA approval, the agency recognizes that the optimal duration of clopidogrel in more complex patients has not been defined. The recommended duration of clopidogrel administration and patient compliance with the prescribed regimen are likely interrelated with patient and anatomical factors that are associated with DES thrombosis. Additional clinical data are likely needed to reach conclusions regarding the optimal antiplatelet therapy regimen for DES patients.

FDA will convene a public meeting of the Circulatory System Devices Advisory Panel by the end of the year in an effort to improve our knowledge regarding the incidence and timing of stent thrombosis as well as the appropriate duration of clopidogrel use in patients who receive DES. This Panel of outside experts will assist the agency in the review and analysis of the available scientific data and provide recommendations for appropriate actions to address this issue, such as possible changes to device labeling or the need for additional clinical studies. An announcement of this meeting will appear on FDA's web site, www.fda.gov/cdrh <http://www.fda.gov/cdrh>.

At this time, FDA believes that coronary DES remain safe and effective when used in patients having clinical and coronary anatomic features similar to those treated in the pivotal trials conducted by the manufacturers for FDA approval. The approved indications are:

- The CYPHER Sirolimus-eluting Coronary Stent is indicated for improving coronary luminal diameter in patients with symptomatic ischemic disease due to discrete *de novo* lesions of length ≤ 30 mm in native coronary arteries with reference vessel diameter of ≥2.5 mm to ≤3.5 mm.

- The TAXUS Express Paclitaxel-Eluting Coronary Stent System is indicated for improving luminal diameter for the treatment of de novo lesions ≤28 mm in length in native coronary arteries ≥2.5 to ≤3.75 mm in diameter.

For more information, see <http://www.fda.gov/cdrh/pdf2/P020026.html> and <http://www.fda.gov/cdrh/pdf3/P030025.html>.

For thousands of patients each year, these devices have resulted in a significant reduction in the need of second procedures to treat restenosis. The FDA will continue to carefully evaluate all DES data in an attempt to maximize the benefits and minimize the risks for patients undergoing this therapy for treatment of their coronary artery disease.

To summarize:

- FDA has been monitoring coronary drug-eluting stents closely since they came on the U.S. market in 2003 and 2004, and will continue to do so.

- New data were released recently that suggest a small but significant increased risk of stent thrombosis in patients who have drug-eluting stents. The agency is keenly interested in this issue because of the potential for serious harm to patients-even though stent thrombosis occurs at low rates.

- While the new data are of interest to FDA and raise important questions, we do not have enough information yet to draw conclusions. It's unclear, for example, what causes drug-eluting stent thrombosis, how often it occurs, under what circumstances it occurs, or what the risk of occurrence is in a given patient.

- To better understand this issue, FDA met with the two manufacturers of these products in recent months to discuss any information they might have pertaining to this issue and get their perspective. In addition, we plan to convene a public panel meeting of outside scientific experts in the near future to assist us in a thorough review of *all* the data and make recommendations about what actions may be appropriate, such as possible labeling changes or additional studies.

At this time, FDA believes that coronary drug-eluting stents remain safe and effective when used for the FDA-approved indications. These devices have significantly reduced the need for a second surgery to treat restenosis for thousands of patients each year.

Appendix 2

Pharmacovigilance

1 Defining Adverse Events

An adverse event (AE) is any untoward medical occurrence in a patient during or following administration of an investigational product and which does not necessarily have a causal relationship with treatment. An AE can therefore be any unfavourable and unintended sign (including an abnormal laboratory finding), symptom, or disease temporarily associated with the use of the trial drugs, whether or not considered related to the trial drugs.

2 Defining Serious Adverse Events (SAEs)

A Serious Adverse Event is defined in general as an untoward (unfavourable) event, associated with trial drug or trial procedure, which:

- is fatal. Death may occur as a result of the basic disease process. Nevertheless, all deaths occurring within 30 days of the last administration of the study agent must be treated as an SAE and reported as such. All deaths which may be considered as related to the trial agent, regardless of the interval, must be treated as a SAE and reported as such.
- is life-threatening
- requires or prolongs hospitalisation
- results in persistent or significant disability or incapacity
- is a congenital anomaly or a birth defect, or
- may require medical or surgical intervention to prevent one of the outcomes listed above
- Any other significant clinical event, not falling into any of the criteria above, but which in the opinion of the investigator requires reporting.

3 Defining Suspected Unexpected Serious Adverse Reactions (SUSARs)

All SAEs assigned by the local investigator as both suspected to be related to the trial drugs and unexpected are subject to expedited reporting. An event is unexpected when information is not consistent with the available product information or investigator brochure, or if they add significant information on the specificity or severity of an expected reaction

4 Reporting AEs

AEs will be collected for all patients. AEs will be collected for all patients from first dose of protocol treatment until 30 days after the last dose of treatment with a protocol IMP.

Information about AEs, whether volunteered by the patient, discovered by the investigator questioning or detected through physical examination, laboratory test or other investigation will be collected and recorded in the study files.

If requested, details of all AEs will be made available after completion of the study.

5 Reporting SAEs

SAEs will be collected for all patients beginning with informed consent. Serious ADRs, which are SAEs considered to be drug-related (at least possibly related), must be reported to the MAH regardless of the time that has elapsed since the end of the trial.

6 Reporting SUSARs

All SAEs assigned by the local investigator as both suspected to be related to study protocol (treatment/procedures) and unexpected (see definition in section 3) will be reviewed by the Principal Investigators (PIs).

Such SAEs will be classified as SUSARs and will be subject to expedited reporting to concerned ethic committees (EC) and regulatory authorities (RA) according to definitions and timelines specified in the local laws and regulations and according to any specific requests made by regulatory authorities.

As a general guideline the following requirements should be used:

- SUSARs must be reported to the EC / RA within 7 calendar days of the PI (or their research team) being informed of the event, if they result in Death or are deemed to be life-threatening.

- Any SUSARs not resulting in Death or deemed to be life-threatening must be reported to the EC / RA within 15 Calendar days of the PI (or their research team) being informed of the event.

In addition, the sponsor shall inform all participating investigators of findings that could adversely affect the safety of study subjects. The information can be aggregated in a line listing of SUSARs in periods as warranted by the nature of the clinical development project and the volume of SUSARs generated. This line listing should be accompanied by a concise summary of the evolving safety profile of the investigational medicinal product.

All SUSARs occurring whilst on trial (until 30 days after the last day of the last treatment) must be reported.

References:

1. Kaiser C, Brunner-La Rocca HP, Buser PT, Bonetti PO, Osswald S, Linka A, Bernheim A, Zutter A, Grize L, Pfisterer ME for the BASKET Investigators. Incremental cost-effectiveness of drug-eluting stents compared with a third-generation bare-metal stent in a real-world setting: randomised Basel Stent Kosten Effektivitats Trial (BASKET). *Lancet*. 2005;366:921-929.

2. Kaiser C,Brunner-La Rocca HP, Buser P, Rickenbacher P, Hunziker P, Jeger R, Mueller C, Pfisterer M for the BASKET Investigators. Long-term cost-effectiveness of drug-eluting stents versus bare-metal stents in a real world setting: 18 months results of the Basel Stent Kosten Effektivitaets Trial (BASKET). Eur Heart J 2006;27, Abstract Supplement.

3. Pfisterer M, Brunner-La Rocca HP, Buser PT, Rickenbacher P, Hunziker P, Mueller C, Jeger R, Bader F, Osswald S, Kaiser C for the BASKET-LATE Investigators. Late Clinical Events after Clopidogrel Discontinuation May Limit the Benefit of Drug-Eluting Stents: An Observational Study of Drug-Eluting vs. Bare-Metal Stents. JACC 2006;48:2584-91

4. Pfisterer M, Brunner-La Rocca HP, Rickenbacher P, Hunziker P, Mueller C, Nietlispach F, Leibundgut G, Bader F and Kaiser C for the BASKET Investigators. Long-term benefit–risk balance of drug-eluting vs. bare-metal stents in daily practice: does stent diameter matter? Three-year follow-up of BASKET. Eur Heart J 2009; 30:16-24.

5. Daemen J, Wenaweser P, Tsuchida K, Abrecht L, Vaina S, Morger C, Kukreja N, Jüni P, Sianos G, Hellige G, van Domburg RT, Hess OM, et al. Early and late coronary stent

thrombosis of sirolimus-eluting and paclitaxel-eluting stents in routine clinical practice: data from a large two-institutional cohort study. Lancet 2007; 369(9562):667-78.0

6. Hans-Peter Brunner-La Rocca, MD, Christoph Kaiser, MD, Matthias Pfisterer, MD for the BASKET Investigators. Targeted Stent Use in Clinical Practice Based on Evidence from the BAsel Stent Cost Effectiveness Trial (BASKET). Eur Heart J 2007; 28:719-25

7. Schofer J, Schluter M, Gershlick AH, Wijns W, Garcia E, Schampaert E, Breithardt G; E-SIRIUS Investigators. Sirolimus-eluting stents for treatment of patients with long atherosclerotic lesions in small coronary arteries: double-blind, randomised controlled trial (E-SIRIUS). Lancet. 2003;362:1093-1099.

8. Degertekin M, Arampatzis CA, Lemos PA, Saia F, Hoye A, Daemen J, Tanabe K, Lee CH, Hofma SJ, Sianos G, McFadden E, van der Giessen W, Smith PC, de Feyter PJ, van Domburg RT, Serruys PW. Very long sirolimus-eluting stent implantation for de novo coronary lesions. Am J Cardiol. 2004;93:826-829.

9. Meier B, Sousa E, Guagliumi G, Van den Branden F, Grenadier E, Windecker S, te Riele H, Voudris V, Eltchaninoff H, Lindvall B, Snead D, Talen A; SVELTE Study Group. Sirolimus-eluting coronary stents in small vessels. Am Heart J. 2006;151:1019.e1-7.

10. Schampaert E, Cohen EA, Schluter M, Reeves F, Traboulsi M, Title LM, Kuntz RE, Popma JJ; C-SIRIUS Investigators. The Canadian study of the sirolimus-eluting stent in the treatment of patients with long de novo lesions in small native coronary arteries (C-SIRIUS). J Am Coll Cardiol. 2004;43:1110-1115.

11. Ardissino D, Cavallini C, Bramucci E, Idolfi C, Marzocchi A, Manari A, Angeloni G, Carosio G, Bonizzoni E, Colusso S, Repetto M, Merlini PA. Sirolimus-eluting vs uncoated

stents for prevention of restenosis in small coronary arteries: a randomized trial. JAMA. 2004;292:2727-2734.

12. Abizaid A, Costa MA, Blanchard D, Albertal M, Eltchaninoff H, Guagliumi G, Geert-Jan L, Abizaid AS, Sousa AG, Wuelfert E, Wietze L, Sousa JE, Serruys PW, Morice MC; Ravel Investigators. Sirolimus-eluting stents inhibit neointimal hyperplasia in diabetic patients. Insights from the RAVEL Trial. Eur Heart J. 2004;25:107-112.

13. Moussa I, Leon MB, Baim DS, O'Neill WW, Popma JJ, Buchbinder M, Midwall J, Simonton CA, Keim E, Wang P, Kuntz RE, Moses JW. Impact of sirolimus-eluting stents on outcome in diabetic patients: a SIRIUS (SIRolImUS-coated Bx Velocity balloon-expandable stent in the treatment of patients with de novo coronary artery lesions) substudy. Circulation. 2004;109:2273-2278.

14. Hermiller JB, Raizner A, Cannon L, Gurbel PA, Kutcher MA, Wong SC, Russell ME, Ellis SG, Mehran R, Stone GW; TAXUS-IV Investigators. Outcomes with the polymer-based paclitaxel-eluting TAXUS stent in patients with diabetes mellitus: the TAXUS-IV trial. J Am Coll Cardiol. 2005;45:1172-1179.

15. Lee MS, Shah AP, Aragon J, Lee MS, Shah AP, Aragon J, Jamali A, Dohad S, Kar S, Makkar RR. Drug-eluting stenting is superior to bare metal stenting in saphenous vein grafts. Catheter Cardiovasc Interv. 2005;66:507-511.

16. Ge L, Iakovou I, Sangiorgi GM, Chieffo A, Melzi G, Cosgrave J, Montorfano M, Michev I, Airoldi F, Carlino M, Corvaja N, Colombo A et al. Treatment of saphenous vein graft lesions with drug-eluting stents: immediate and midterm outcome. J Am Coll Cardiol. 2005;45:989-994.

17. Pfisterer M, Bertel O, Bonetti PO, MD, Brunner-La Rocca HP, Eberli FR, Erne P, Galatius S, Hornig B, Kiowski W, Pachinger O, Pedrazzini G, et al., for the BASKET-PROVE Investigators. Drug-eluting or bare-metal stents for large coronary vessel stenting? The BASKET-PROVE (PROspective Validation Examination) trial: Study protocol and design. Am Heart J 2008;155:609-14.

18. Joner M, Finn AV, Farb A, Mont EK, Kolodgie FD, Ladich E, Kutys R, Skorija K, Gold HK, Virmani R. Pathology of drug-eluting stents in humans: delayed healing and late thrombotic risk. J Am Coll Cardiol. 2006;48:193-202.

19. Kotani J, Awata M, Nanto S, Uematsu M, Oshima F, Minamiguchi H, Mintz GS, Nagata S. Incomplete neointimal coverage of sirolimus-eluting stents: angioscopic findings. J Am Coll Cardiol. 2006;47:2108-2111.

20. Peters RJ, Mehta SR, Fox KA, Zhao F, Lewis BS, Kopecky SL, Diaz R, Commerford PJ, Valentin V, Yusuf S; Clopidogrel in Unstable angina to prevent Recurrent Events (CURE) Trial Investigators.Effects of aspirin dose when used alone or in combination with clopidogrel in patients with acute coronary syndromes: observations from the Clopidogrel in Unstable angina to prevent Recurrent Events (CURE) study. Circulation 2003;108:1682-7.

21. Mehta SR,Yusuf S, Peters RJ, Bertrand ME, Lewis BS, Natarajan MK, Malmberg K, Rupprecht H, Zhao F, Chrolavicius S, Copland I, Fox KA; Clopidogrel in Unstable angina to prevent Recurrent Events trial (CURE) Investigators. Effects of pretreatment with clopidogrel and aspirin followed by long-term therapy in patients undergoing percutaneous coronary intervention: the PCI-CURE study. Lancet 2001;358:527-33

22. Windecker S, Serruys P, Wandel S, Buszmann P, Trznadel S, Linke A, Ischinger T, Klauss V, Eberli F, Corti R, Wijns W, Morice MC et al. Biolimus-eluting stent with biodegradable polymer versus sirolimus-eluting stent with durable polymer for coronary revacularisation (LEADERS): a randomised non-inferiority trial. Lancet 2008;372:1163-73.

23. Tung R, Kaul S, Diamond GA, Shah PK. Narrative review: Drug-eluting stents for the management of restenosis: a critical appraisal of the evidence. Ann Intern Med 2006;144:913-19.

24. Dahm JB, Willems T, Wolpers HG, Nordbeck H, Becker J, Ruppert J. Clinical investigation into the observation that silicon carbide coating on cobalt chromium stents leads to early differentiating functional endothelial layer, increased safety and DES-like recurrent stenosis rates: results of the PRO-Heal Registry (PRO-Kinetic enhancing rapid in-stent endothelialisation) EuroInterv 2008;4:502-8.

25. Wiviott SD, Braunwald EG, McCabe CH, Horvath I, Keltai M, Herrmann JPR, Van de Werf F, Downey WE, Scirica BM, Murphy SA, Antmann EM for the TRITON-TIMI 38 investigators. Intensive oral antiplatelet therapy for reduction of ischemic events including stent thrombosis in patients with acute coronary syndromes treated with percutaneous coronary intervention and stenting in the TRITON-TIMI 38 trial: a subanalysis of a randomised trial. Lancet 2008;371:1553-63.

26. Greiner W, Claes C, Busschbach JJV, Graf von der Schulenburg J-M. Validating the EQ-5D with time trade off fort he german population. Eur J Health Econom 2005;6:124-30.

27. Greiner W, Weijnen T, Nieuwenhuizen M et al. A single European currency for EQ-5D health states. Eur J Health Econom 2003;4:221-31.

28. Myocardial infarction redefined - a consensus document of The Joint European Society of Cardiology/American College of Cardiology Committee for the redefinition of myocardial infarction. Eur Heart J 2000;21:1502–13.

29. Cutlip DE, Windecker S, Mehran R, Boam A, Cohen DJ, van Es GA, Steg PG, Morel MA, Mauri L, Vranchx P, McFadden E, Lansky A, Hamon M, Krucoff MW, Serruys PW, Academic Research Consortium. Clinical end points in coronary stent trials: A case for standardized definitions. Circulation 2007;115:2344-51

30. Brunner-La Rocca HP, Buser PT, Schindler R, Bernheim A, Rickenbacher P, Pfisterer M for the TIME-CHF Investigators. Management of elderly patients with congestive heart failure – design of the Trial of Intensified versus standard Medical therapy in Elderly patients with Congestive Heart Failure (TIME-CHF). Am Heart J 151; 949-955 (2006)

Abbreviations

ACS	Acute Coronary Syndrome
BMS	Bare Metal Stent
CAD	Coronary Artery Disease
CABG	Coronary Artery Bypass Grafting
CE	European Market Label
CEC	Critical Events Committee
CRF	Case Report Form
DES	Drug Eluting Stent
EQ-5D	European perspective questionnaire
ESC-guideline	European Society of Cardiology
FDA	Food and Drug Administration
GP IIb/IIIa inhibitors	Glycoprotein IIb/IIIa Receptor – Inhibitor
JACC	Journal of American College of Cardiology
MACE	Major Adverse Cardiac Event
MI	Myocardial Infarction
PCI	Percutaneous Coronary Intervention
SPSS 14.0	Statistic Program
TVR	Target Vessel Revascularization
