# Supplementary material for: Competing risks of major bleeding and thrombotic events with prasugrel-based dual antiplatelet therapy after stent implantation - An observational analysis from BASKET-PROVE II
Source: PLoS One. 2019 Jan 15;14(1):e0210821. doi: 10.1371/journal.pone.0210821 (PMC6333357; doi:10.1371/journal.pone.0210821)
Supplement: S1 Protocol Amendment — (PDF) [file pone.0210821.s005.pdf]

Evaluation of late clinical events after drug-eluting versus bare-metal stents in patients at risk: BAsel Stent Kosten Effektivitäts Trial – PROspective Validation - Examination Part II

BASKET-PROVE II

(November 09, 2009)

Amendment III
Safety / Bleeding Analysis

(January 10th, 2012)

Sponsor: Department Cardiology**,** University Hospital Basel, Switzerland

Principal Investigator/Clinical Trial Leader: PD Dr. med. Christoph Kaiser

Medical Expert: PD Dr. med. Christoph Kaiser, University Hospital Basel, Petersgraben 4, 4031 Basel, Switzerland, +41 (0)61 328 72 25

Signature:____________________________ **Date:** __10.01.2012______

Principal Investigator Site:

Name: **Site:**

Signature: _______________________ **Date:** __________________

Safety/Bleeding-Rates of Prasugrel-based Dual Antiplatelet Therapy in Patients with Stable Coronary Artery Disease

1. Background

Prasugrel is approved for use in acute coronary syndrome (ACS) patients undergoing PCI and stent implantation based on the findings of TRITON-TIMI 38 (1) with certain safety precautions: patients with a history of a cerbrovascular event as well as those age >75 years or weighing <60kg should not receive it due to increased bleeding risk. This was implemented in the new US guidelines on percutaneous coronary interventions (PCI) (2) stating that prasugrel-based double antiplatelet therapy (DAPT) should not be used in the first two mentioned groups but could be given to patients with a body weight <60kg in a reduced dose. In patients with stable coronary artery disease (CAD), corresponding trial safety data is lacking.

According to the approved study protocol of BASKET PROVE II (BP II(3)), all patients presenting with ACS as well as stable CAD are treated in this study with aspirin and prasugrel except for those with a history of a cerebrovascular event (excluded from BP II); patients >75 years of age and those weighing <60kg are treated with 5mg instead of 10mg prasugrel daily. Prasugrel-based DAPT is prescribed for 12 months in all patients with drug-eluting stents (DES) and for ACS patients with bare-metal stents (BMS), while stable patients with BMS are treated for only 1 month. – This treatment scheme asks for a specific safety surveillance and analysis regarding bleeding in patients included for stable CAD. This has been

implemented with particular monitoring of bleeding events and a secondary analysis comparing bleeding in BP II (with prasugrel-based DAPT) with bleeding events observed in BASKET PROVE (BP (4); with a clopidogrel-based DAPT).

Different definitions of bleeding events have been used in different trials (5): the TIMI and the GUSTO definitions primarily used in fibrinolytic trials and, more recently, the PLATO and other definitions for stent trials. The Academic Research Consortium has therefore convened to define bleeding events consistently for future stent trials (6): the BARC classification (see Table Appendix).

This Amendment III extends and specifies the safety analysis with regard to bleeding events, proposed in the main protocol of BP II, including Amendment II.

2. Aim of the safety/bleeding analysis of BP II

Since there are no safety data from randomised clinical trials regarding bleeding on Clopidogrel- versus Prasugrel-based DAPT in patients with stable CAD, as opposed to patients with ACS, the primary aim of this analysis will be

- **to document the safety – or possible harm – of DAPT with prasugrel and aspirin in patients with stable CAD regarding bleeding events.**

This will be achieved in two ways:

A. Within BP II, bleeding rates of patients with stable CAD will be compared with bleeding rates of patients presenting with ACS
B. The bleeding rates observed in BP II (DAPT with prasugrel and aspirin) will be compared with bleeding rates of the predecessor study BASKET PROVE (DAPT with clopidogrel and aspirin). This comparison will include patients with all indications allowing to compare bleeding rates of patients with stable CAD and ACS between trials (and type of DAPT).

In BASKET PROVE (BP), DAPT was given as clopidogrel and aspirin for 12 months in all patients, independent of their indication and stent type used. In BP II, DAPT with prasugrel and aspirin was reduced to 1 month in BMS patients with stable CAD. The planned comparisons will therefore focus on patients with DES and 12 months DAPT, but consider all patients of both trials irrespective of indication.

3. Methods

As specified in the main protocol (3), all bleeding events will be recorded in the CRF with special monitoring (source documentation) in order to apply both the TIMI and the BARC criteria for bleeding. All bleeding events will be adjudicated by a Critical Events Committee blinded to the stent treatment assignment. All bleeding events of the prior BP study already adjudicated according to the TIMI criteria will be reassessed according to the BARC criteria.

“Any relevant” non-CABG related bleeding will be defined as TIMI minor/major or BARC types 2-4 and 6, whereas “severe” bleeding will be TIMI major or BARC types 3,4 or 6.

3.1 Patient populations

A. **Stable vs. ACS within BP II:** Expected numbers of patients in BP II are shown in Table 1. The comparison will include bleeding events of all DES patients with 12 months DAPT, i.e., 560 stable CAD patients versus 1040 ACS patients.

Table 1: Expected patient numbers in BP II. The study is ongoing, a total of 2400 patients will be recruited. Approximately 35% will present with CAD and 65% with ACS.

	BMS	DES	Total
Stable	280	560	840
ACS	520	1040	1560
Total	800	1600	2400

If, as expected and previously shown (1), there will be no differences in bleeding rates between patients receiving DES and BMS and DAPT for 12 months within BP II, bleeding rates of stable CAD patients with 12 months DAPT (within BP II, n = 560) will also be compared with those of ALL ACS patients with 12 months DAPT (within BP II, n = 520 +1040 = 1560).

B. Stable and ACS in BP II vs. BP: The two studies (BP II and BP) have the same size with about 2400 patients randomized 2:1 to DES vs. BMS. Table 2 shows patient numbers from BP.
A first comparison, focussing on the difference between prasugrel and clopidogrel, will include bleeding events of all DES patients with 1 year DAPT (stable CAD and ACS), i.e., 1600 BP II patients with prasugrel vs. 1549 BP patients with clopidogrel.

A second comparison, focusing on the difference between 1 month DAPT with prasugrel and 1 year DAPT with clopidogrel (different treatment duration and different drugs) in stable CAD patients, will include bleeding events of all BMS patients with stable CAD, i.e. 280 BPII patients with 1 month prasugrel versus 285 BP patients with 1 year clopidogrel.

Table 2: Patient numbers in BP.

	BMS	DES	Total
Stable	285	537	822
ACS	480	1012	1492
Total	765	1549	2314

3.2 Statistical analyses

Baseline Comparisons

Baseline parameters will be compared between the two indication groups (stable CAD and ACS patients receiving a DES) within BP II (and BP), as well as between the two studies (BP and BP II).

A. Stable vs. ACS within BP II

The endpoint TIMI major (or BARC 3,4 or 6) bleeding events will be compared between stable and ACS patients in BP II (only those receiving a DES) using a Cox-proportional hazards survival model. Although BP II is a randomized trial (randomized for stent type), “indication" (stable vs. ACS) is an observational variable, likely to be confounded with other patient characteristics. To balance the data for confounding effects, a *propensity score* weighted analysis will be used (7). The predictors for the calculation of propensity scores will include potential confounders, i.e.,

variables that may affect the indication as well as the outcome (occurrence of bleeding events) such as: age, sex, body weight, renal function, hypertension, diabetes, prior MI, as well as all baseline variables where the two patient groups (stable CAD vs. ACS) will be shown to differ. We will use *inverse probability weighting* (8) to estimate the effect of the “indication" on the hazard of bleeding.

The survival model will include the “indication" (stable CAD vs. ACS) as predictor. Further predictors in the model will be the “dosage of prasugrel" (since patients older than 75 years and weighing less than 60 kg receive only half of the dosage) and “use of GP 2b/3a blockers". The use of GP 2b/3a blockers is typically much more frequent in ACS patients (33.1% in BP) than in stable CAD patients (2.6% in BP), and is known to increase the occurrence of early bleeding events.

B. **Stable and ACS in BP II vs. BP**

The endpoint TIMI major (or BARC 3,4 or 6) bleeding event will be compared between DES patients in BP II and DES patients in BP using a Cox-proportional hazards survival model. Because patient populations come from two different trials rather than from one single randomised trial, and because the “indication” may be confounded with other patient characteristics (as for Analysis A), a *propensity score* weighted analysis will be used again (see above) to estimate the effects of interest. The survival model will include “DAPT" (prasugrel vs. clopidogrel) and “indication" (stable CAD vs. ACS) as predictors. In addition, the model will include the interaction between DAPT and indication (stable CAD versus ACS), to test whether the difference due to the type of DAPT depends on the indication.

General

To add a time-independent analysis, particularly for the case of non-proportional hazards, the survival analysis will be complemented by logistic regression on bleeding events at 12 months, estimating odds ratios instead of hazard ratios.

All statistical analyses will be performed by an independent statistician at the Clinical Trial Unit at the University Hospital in Basel, Switzerland, using the statistical software package R (9).

4. Approval of this Amendment

This amendment will be submitted for approval to the local ethical committees of each participating center. Since it specifies the safety analysis of trial data only, no separate informed patient consent is necessary. This amendment will also be registred together with the already registred BP II trial (NCT01166685).

5. Relevance of this analysis

If the 2-year rates of DES patients presenting with stable CAD treated with aspirin and prasugrel for 1 year are not significantly different from those of DES patients presenting with ACS and not significantly higher than in DES patients of the predecessor BP trial treated for 1 year with aspirin and clopidogrel, then this would suggest that *“a prasugrel-based DAPT may be as safe in stable CAD patients as in ACS patients” (where it is*

approved); if above assumptions will prove wrong, then such a conclusion would not be allowed. – In any case, such data will have to be confirmed in a direct comparison in a large randomized controlled trial. Still, the proposed analysis will be an important first step in the evaluation of the safety of a prasugrel-based DAPT in stable CAD patients.

6. References

1. Wiviott SD, Braunwald E, McCabe CH, Montalescot G, Ruzyllo W, Gottlieb S, Neumann FJ, Ardissino D, DeServi S, Murphy SA, Riesmeyer J, Weerakkody G, Gibson CM, Antmann EM. Prasugrel versus clopidogrel in patients with acute coronary syndromes. N Engl J Med 2007;357:2001-15.
2. Levine GL, Bates ER, Blankenship JC, Bailey SR, Bittl JA, Cercek B, Chambers CE, Ellis SG, Guyten RA, Hollenberg SM, Khot UN, Lange RA, Mauri L, Mehran R, Moussa ID, Mukherjee D, Brahmajee K. 2011 ACCF/AHA/SCAI Guideline for Percutaneous Coronary Intervention. J Am Coll Cardiol 2011, online
3. Jeger R, Pfisterer M, Alber H, Eberli F, Galatius S, Naber C, Pedrazzini G, Rickli H, Skov Jensen J, Vuilliomenet A, Gilgen N,[1] and Kaiser C, for the BASKET-PROVE II Investigators. Newest-Generation Drug-Eluting and Bare-Metal Stents Combined With Prasugrel-based Antiplatelet Therapy in Large Coronary Arteries: The BAsel Stent Kosten Effektivitäts Trial PROspective Validation Examination part II (BASKET-PROVE II) – Trial Design.Am Heart J, 2011 (in press)
4. Kaiser C, Galatius S, Erne P, Eberli F, Alber H, Rickli H, Pedrazzini G, Hornig B, Bertel O, Bonetti P, Brunner-LaRocca HP, Richard I, Pfisterer M. Drug-eluting versus bare-metal stents in large coronary arteries. New Engl J Med 2010;363:2310-9.
5. Steg PG, Huber K, Andreotti F, Arnesen H, Atar D, Badimon L, Bassand JP, DeCaterina R, Eikelboom JA, Gulba D, Hamon M, Helft G, Fox KAA, Kistensen SD, Rao SV, Verheught FWA, Widimsky P, Zeymer U, Collet JP. Bleeding in acute coronary syndromes and percutaneous interventions: position paper by the Working Group on

Thrombosis of the European Spociety of Cardiology. Eur Heart J 2011;32:1854-64.

6. Mehran R, Rao SV, Bhatt DL, Gibson CM, Caixeta A, Eikelboom J, Kaul S, Wiviott SD, Menon V, Nikolsky E, Serebruany V, Valgimigli M, Vranckx P, Taggart D, Sabik JF, Cutlip DE, Krucoff MW, Ohman EM, Steg P, White H. Standardized Bleeding Definitions for Cardiovascular Clinical Trials: A Consensus Report from the Bleeding Academic Research Consortium (BARC). Circulation 2011;123:2736–2747.
7. Tibshirani, R. 1996: Regression shrinkage and selection via the lasso. Journal of the Royal Statistical Society. Series B (Methodological) 58:267-88.
8. Hernan, M. A. & Robins, J. M. 2006: Estimating causal e_ects from epidemiological data. J Epidemiol Community Health 60:578-86.
9. R Development Core Team. 2011: R: A language and environment for statistical computing. R Foundation for Statistical Computing, Vienna, Austria.

Appendix Table: BARC definition for bleeding (5,7)

Type 0: No bleeding

Type 1: Bleeding that is not actionable and does not cause the patient to seek unscheduled performance of studies, hospitalization, or treatment by a health care professional

Type 2: Any overt, actionable sign of haemorrhage (e.g. more bleeding than would be expected for a clinical circumstance; including bleeding found by imaging alone) that does not fit the criteria for Types 3, 4, or 5, but does meet at least one of the following criteria:

(1) requiring non-surgical, medical intervention by a health care professional,

(2) leading to hospitalization or increased level of care,

(3) prompting evaluation

Type 3

Type **3a**

Overt bleeding plus haemoglobin drop of 3 to ,5*g/dL (provided haemoglobin drop is related to bleed)

Any transfusion with overt bleeding

Type **3b**

Overt bleeding plus haemoglobin drop ≥ 5*g/dL (provided haemoglobin drop is related to bleed)

Cardiac tamponade

Bleeding requiring surgical intervention for control (excluding dental/nasal/skin/haemorrhoid)

Bleeding requiring intravenous vasoactive drugs

Type **3c**

Intracranial haemorrhage (does not include microbleeds or haemorrhagic transformation; does include intraspinal)

Subcategories; confirmed by autopsy or imaging or LP

Intra-ocular bleed compromising vision

Type 4: CABG-related bleeding

Perioperative intracranial bleeding within 48 h

Reoperation following closure of sternotomy for the purpose of controlling bleeding

Transfusion of ≥ 5 units of whole blood or packed red blood cells within a 48 period**
Chest tube output ≥ 2 L within a 24 h period
If a CABG-related bleed is not adjudicated as at least a Type 3 severity event, it will be classified as ‘not a bleeding event’

Type 5: fatal bleeding
Type **5a**
Probable fatal bleeding: no autopsy or imaging confirmation, but clinically suspicious
Type **5b**
Definite fatal bleeding: overt bleeding or autopsy or imaging confirmation
Obs: Platelet transfusions should be recorded and reported, but are not included in these definitions until further information is obtained about the relationship to outcomes.

---

*Corrected for transfusion (1 unit PRBC or 1 unit whole blood ¼ 1 g/dL Hgb). **Only allogeneic transfusions are considered transfusions for BARC Type 4 bleeding. Cell saver
products will not be counted.

Approved by the Steering Committee on January 10, 2012
